# Proximity-Induced Exchange Interaction and Prolonged Valley Lifetime in MoSe_2_/CrSBr Van-Der-Waals Heterostructure with Orthogonal Spin Textures

**DOI:** 10.1021/acsnano.4c07336

**Published:** 2024-10-28

**Authors:** Andreas Beer, Klaus Zollner, Caique Serati de Brito, Paulo E. Faria Junior, Philipp Parzefall, Talieh S. Ghiasi, Josep Ingla-Aynés, Samuel Mañas-Valero, Carla Boix-Constant, Kenji Watanabe, Takashi Taniguchi, Jaroslav Fabian, Herre S. J. van der Zant, Yara Galvão Gobato, Christian Schüller

**Affiliations:** † Institut für Experimentelle und Angewandte Physik, Universität Regensburg, D-93040 Regensburg, Germany; ‡ Institute of Theoretical Physics, University of Regensburg, 93040 Regensburg, Germany; § Physics Department, Federal University of São Carlos, São Carlos, SP 13565-905, Brazil; ∥ Kavli Institute of Nanoscience, Delft University of Technology, Lorentzweg 1, 2628 CJ Delft, The Netherlands; ⊥ Instituto de Ciencia Molecular (ICMol), Universitat de València, Catedrático José Beltrán 2, Paterna 46980, Spain; # Research Center for Materials Nanoarchitectonics, National Institute for Materials Science, 1-1 Namiki, Tsukuba 305-0044, Japan; ## Research Center for Electronic and Optical Materials, National Institute for Materials Science, 1-1 Namiki, Tsukuba 305-0044, Japan

**Keywords:** transition metal dichalcogenides, two-dimensional magnets, van der Waals heterostructures, optical spectroscopy, time-resolved Kerr rotation

## Abstract

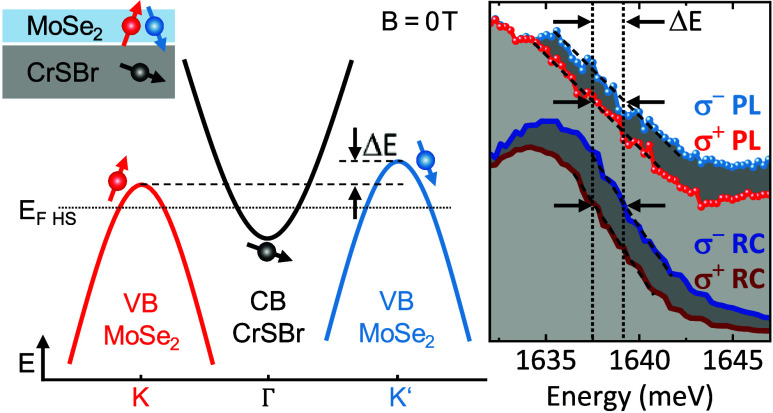

Heterostructures, composed of semiconducting transition-metal dichalcogenides (TMDC) and magnetic van-der-Waals materials, offer exciting prospects for the manipulation of the TMDC valley properties via proximity interaction with the magnetic material. We show that the atomic proximity of monolayer MoSe_2_ and the antiferromagnetic van-der-Waals crystal CrSBr leads to an unexpected breaking of time-reversal symmetry, with originally perpendicular spin directions in both materials. The observed effect can be traced back to a proximity-induced exchange interaction via first-principles calculations. The resulting spin splitting in MoSe_2_ is determined experimentally and theoretically to be on the order of a few meV. Moreover, we find a more than 2 orders of magnitude longer valley lifetime of spin-polarized charge carriers in the heterostructure, as compared to monolayer MoSe_2_/SiO_2_, driven by a Mott transition in the type-III band-aligned heterostructure.

Van-der-Waals (vdW) heterostructures offer the outstanding possibility of combining atomically thin layers of different material classes, independent of their specific crystal structures. Recent studies on monolayer (ML) transition-metal dichalcogenides (TMDCs) and magnetic vdW materials have mostly concentrated on ferromagnetic materials with out-of-plane magnetization.^1−7^ However, there are emergent vdW magnetic materials such as CrSBr, which have some particularly appealing properties.^8^ In the bulk form, CrSBr represents electronically a quasi-one-dimensional semiconductor,^9^ and, the interlayer electronic coupling can be controlled by the layered magnetic order.^10^ CrSBr is a layered semiconductor with a rectangular unit cell in the plane (**â** and **b̂** axes) and orthorhombic bulk structure (perpendicular **ĉ** axis). Interestingly, single layers are ferromagnetic with in-plane magnetization, while the coupling between layers is antiferromagnetic.^10^ Bulk material is known to be an A-type antiferromagnet with a Neél temperature of 132 K,^9−17^ with easy and intermediate magnetic axes along **b̂** and **â** axes, respectively, and a hard axis along **ĉ**.^18^ Another phase transition around *T* = 40 K was identified in CrSBr, which might be related to crystal defects^8,11^ or spin-freezing effects.^14,19^ The highly anisotropic electronic and magnetic structure of CrSBr directly reflects on its optical properties: Changes in the static magnetic configuration of CrSBr by the application of a magnetic field directly impact the exciton energy. This can be used to probe its magnetic properties.^10,11,14,20^ Alternatively, the temperature-dependent magnetic phases can be monitored by Raman spectroscopy.^21^ Importantly, the strongly anisotropic electronic band structure favors optical absorption for light polarized parallel to the **b̂** axis, while absorption is strongly suppressed for perpendicular polarization, along the **â** axis.^9,10^ Coupling of excitons and coherent magnons in CrSBr was first demonstrated in refs (17) and (22). On the other hand, the semiconducting ML-TMDCs have a hexagonal crystal lattice, and intriguing properties, like large exciton binding energies,^23,24^ large oscillator strength,^25^ valley-contrasting optical selection rules,^26^ and spin-valley locking.^27^ Very recently, first hybrid structures of CrSBr and ML-TMDCs have been demonstrated and revealed intriguing properties.^28^

In this article, a ML-MoSe_2_/CrSBr heterostructure is explored via time-resolved optical spectroscopy in combination with *ab initio* band structure calculations, based on density-functional theory (DFT). In photoluminescence (PL), reflectance contrast (RC), and time-resolved Kerr ellipticity (TRKE) experiments, we find evidence for a breaking of time-reversal symmetry in the MoSe_2_ layer of the heterostructure. This is surprising since the spin exchange field in CrSBr and the spin–orbit field in ML-MoSe_2_ are originally orthogonal. We can trace back our observations to a proximity-induced exchange interaction, found by DFT band structure calculations. Furthermore, we find a more than 2 orders of magnitude longer valley lifetime in the heterostructure, as compared to plain MoSe_2_ monolayers.

## Results and Discussion

### Sample Characterization and Quasi-Static Experiments

**Figure 1:**
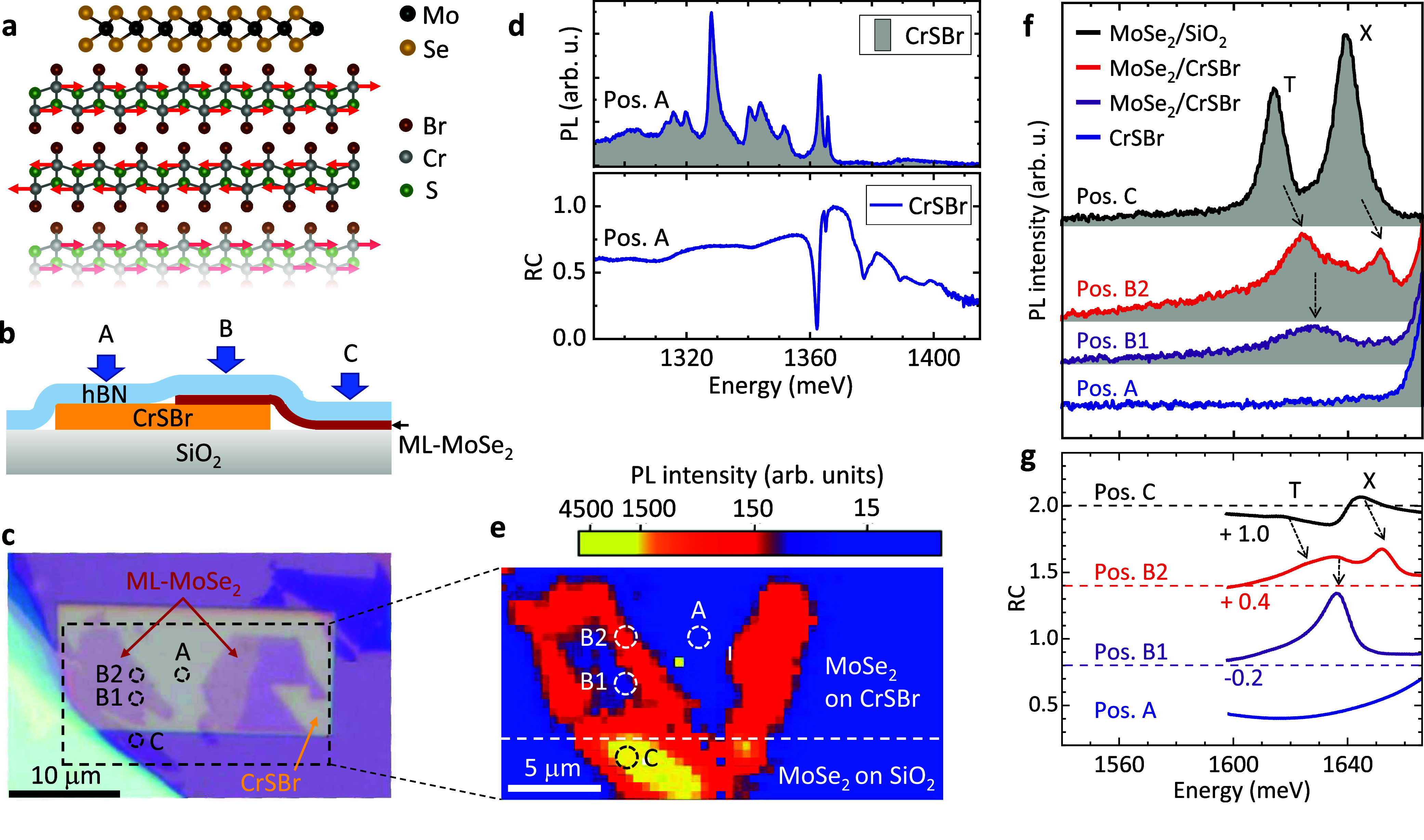
ML-MoSe_2_/CrSBr sample, PL and RC measurements. (a) Schematic picture of the MoSe_2_–CrSBr vdW heterostructure. The in-plane spins in CrSBr (red arrows along the **b** easy axis) are located at the Cr ions. (b) Cross-sectional schematic drawing of the investigated sample. Three different characteristic areas of the sample are labeled by the letters A, B, and C. (c) Microscopy image of the sample. Selected measurement spots are indicated. Note that the hBN covering layer is larger than the picture frame. Therefore, it is not visible. (d) PL and RC spectra of the CrSBr bulk part in sample area A. (e) PL intensity plot of the area, outlined by dashed lines in (c). Selected measurement spots are indicated. Note the logarithmic intensity scale (in the PL scan, *T* = 4 K). (f) Comparison of PL spectra, taken at the positions marked in (c, e). (g) RC spectra taken at the same sample positions as in (f). The zero lines are indicated by dashed lines of the same color. For the measurements in (f, g), the temperature was *T* = 15 K.

Figure 1a,1b shows a schematic picture of the ML-MoSe_2_/CrSBr vdW heterostructure and a cross-sectional drawing of the investigated sample, respectively. There are three distinct sample areas, labeled by A-C, namely, bulk CrSBr, the ML-MoSe_2_/CrSBr heterostructure, and ML-MoSe_2_ on SiO_2_, respectively. The whole sample is covered by a large, about 20 nm thick hexagonal Boron Nitride (hBN) layer. Characteristic positions are indicated in the microscope image in Figure 1c. An intensity plot of the MoSe_2_ PL, recorded within the outlined area in Figure 1c, is displayed in Figure 1e. Representative PL and RC spectra are shown in Figure 1f,1g, respectively. On position C, the well-known PL of the neutral exciton (X) and trion (T) of ML-MoSe_2_ is observed.^23^ This is by far the brightest PL signal of the ML region of the sample (cf. Figure 1e). The faintest ML PL is observed in the region of position B1. There, the two materials are in optimal contact; i.e., the ML PL is strongly quenched (purple spectrum in Figure 1f). Nevertheless, this region shows a strong oscillator in the RC (purple spectrum in Figure 1g), located at the high-energy end of the corresponding PL. The intermediate contact region of position B2 is anticipated to have a less perfect contact. Therefore, the excitonic MoSe_2_ PL is not completely quenched (red spectrum in Figure 1f). On bulk CrSBr (position A), there is no distinct PL transition observable in this spectral window, and the corresponding RC spectrum is also featureless. This is different in the energetic region below 1360 meV, where the well-known strong excitonic/polaritonic transitions of bulk CrSBr^9,28,29^ can be observed in PL and RC spectra (upper and lower panel of Figure 1d).

**Figure 2:**
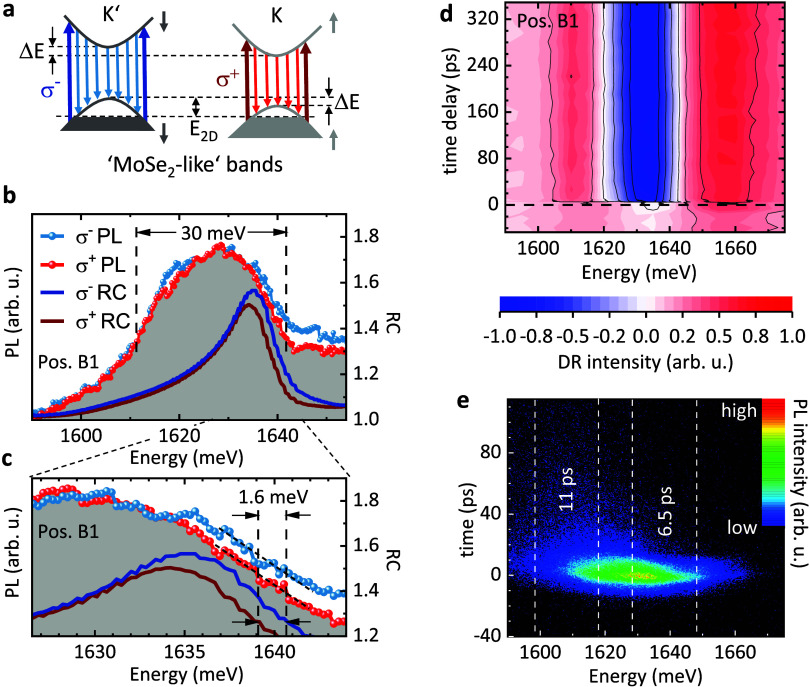
Circularly polarized PL, RC, and two-color pump–probe experiments on ML-MoSe_2_/CrSBr, spot B1. (a) Schematic picture of the MoSe_2_-like bands of the heterostructure. The proximity-induced splitting Δ*E* is indicated. Transitions between the states of the heterostructure, contributing to the PL (light blue and red arrows), and absorption at the Fermi edge (dark blue and red arrows) are given. (b) Circularly polarized PL and RC spectra of the MoSe_2_/CrSBr heterostructure. (c) Zoom into the high-energy flanks of the PL and RC spectra displayed in panel (b). (d) Intensity plot of differential reflectivity (DR) time traces for different probe-energy windows of 13 meV spectral widths. The pump energy is centered at 1690 meV. Pump- and probe pulses are linearly polarized. (e) Streak-camera image of time-resolved PL of the heterostructure.

It was already anticipated in ref (28) that ML-MoSe_2_/CrSBr forms a type-III band alignment, resulting in a p-type doping of MoSe_2_. We will foster this below by first-principles band structure calculations. Moreover, our calculations show that there is a proximity-induced spin splitting Δ*E* of the MoSe_2_ valence and conduction bands. This scenario is depicted qualitatively in Figure 2a, where the “MoSe_2_-like” bands of the heterostructure are drawn with a valley splitting Δ*E*. From our PL spectra in the region of position B1, we infer that we are already in the Mott regime (see ref (30)), where excitonic interaction is largely screened, and the broad PL is caused by band-to-band recombination. Due to counteracting energy shifts - caused by bandgap renormalization on the one hand, and reduction of the exciton binding energy with increasing density of free carriers on the other hand - the band-to-band transition in the Mott regime is approximately at the same energy as the excitonic transition in the dilute regime.^30,31^ We analyze this experimentally further by the enlarged plot of the polarization-resolved heterostructure PL in Figure 2b, under the assumption that the PL line width is determined by the energetic spread of the light blue and red arrows in Figure 2a. Accordingly, the experimental PL line width of ∼30 meV equals approximately 2E_2D_ (cf. Figure 2a). With effective electron and hole masses of m* ∼ 0.6 m_0_,^32^ we get a hole density of *p* ∼ 3.8 × 10^12^ cm^–2^. We note that the total density of free holes and electrons at the heterostructure interface is twice as large due to charge neutrality, i.e., *n*_total_ ∼ 7.6 × 10^12^ cm^–2^. In ref (33), Mott densities in the range 10^12^–10^14^ cm^–2^, depending on the pump energy, were found in nonlinear optical experiments on MoSe_2_ monolayers. Calculations in ref (30) revealed similar Mott densities for MoS_2_ and MoSe_2_. The reported critical density^34^ of MoS_2_ is 5 × 10^12^ cm^–2^, which is below our total density of free charge carriers. We further note that, since the Mott transition in TMDCs is accompanied by a rather delicate mixture of exciton and free-electron phases,^30^ we can not definitely exclude that trions, or Fermi polarons,^35^ still play a role in our experiments. The high oscillator strengths in the RC spectra at an energy of ∼1640 meV (dark blue and red curves in Figure 2b) might be due to a Fermi-edge-singularity effect in absorption.^34,36−41^

Experimental evidence of a proximity-induced spin splitting of the MoSe_2_ layer can be obtained from the circularly polarized PL and RC spectra in Figure 2b. Figure 2c shows a zoomed-in view of their high-energy flanks. We observe consistently in both PL and RC, a splitting of ∼1.6 meV between the high-energy ends of the σ^+^- and σ^–^-polarized spectra. This can be explained qualitatively by the energetic differences of the blue and red arrows in Figure 2a, due to different valley populations (light colors for PL and dark colors for RC).

### Time-Resolved Optical Experiments

For finding the best parameters for pump–probe experiments on the heterostructure, we first tested the probe-energy dependence of the differential reflectivity (DR) signal. Figure 2d shows an intensity plot of a series of DR traces for different, equally spaced probe-energy windows of 13 meV width, taken with linearly polarized light. The energy of the pump pulses is kept fixed at an energy of 1690 meV, i.e., above the optical bandgap. We get a negative and maximal DR response for probe energies of ∼1633 meV, i.e., below the Fermi-edge transition at ∼1640 meV, and a weaker, positive DR response at energies above 1640 meV. Based on these findings, we fix the probe-energy window for the subsequent experiments on the heterostructure to a center energy of ∼1633 meV to get maximal responses.

**Figure 3:**
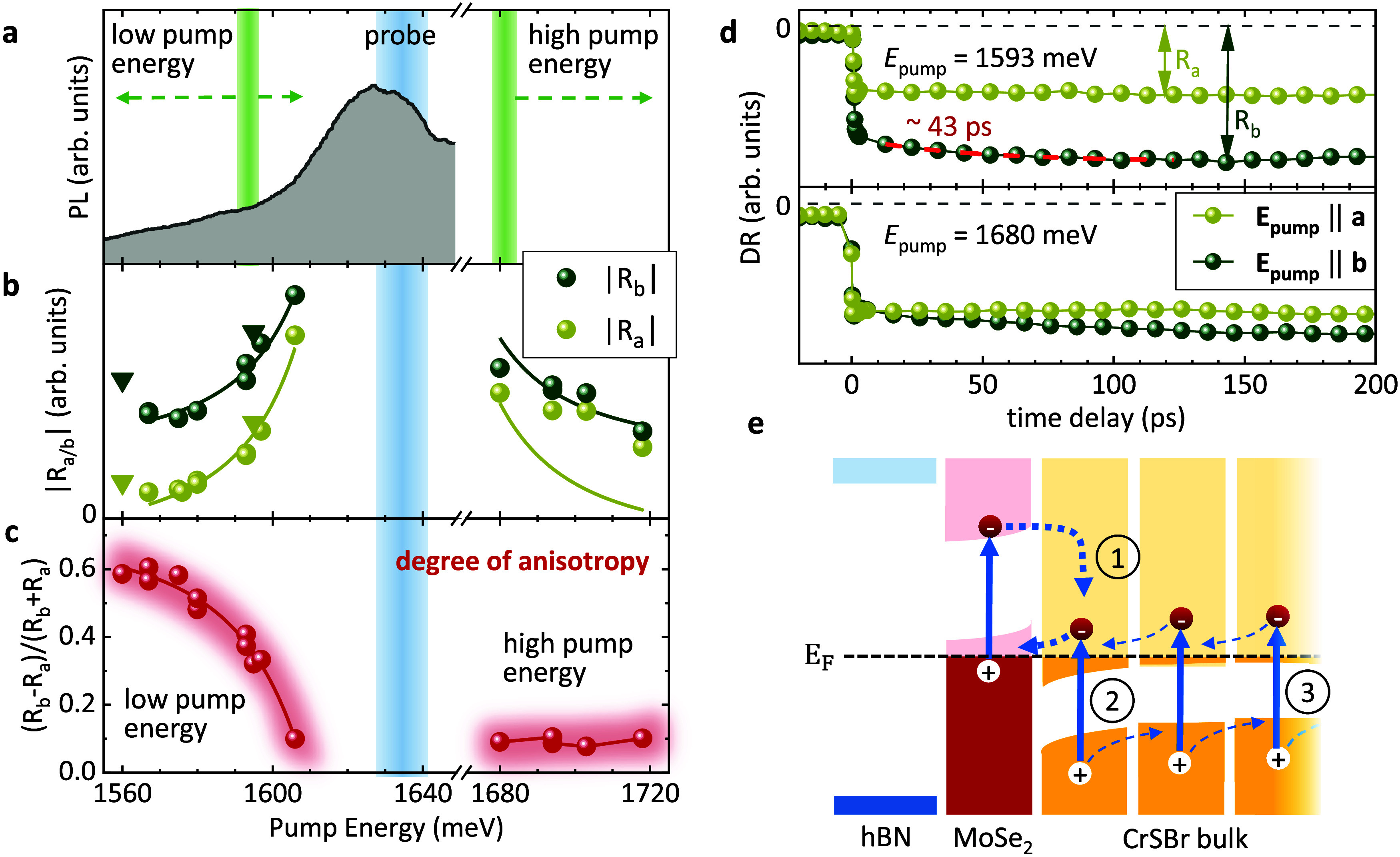
Two-color DR experiments on ML-MoSe_2_/CrSBr heterostructure, spot B1. (a) PL spectrum of the heterostructure region with optimal contact. Spectral ranges of pump and probe pulses are indicated. The pump pulses are tuned either to lower or to higher energy with respect to the probe pulses. (b) Amplitudes of the DR signal versus pump energy. Triangles are data points from a different measurement series for verification. (c) Evaluation of the degree of anisotropy with respect to the two in-plane axes, **â** and **b̂**. (d) DR time traces for pump/probe pulses as indicated in (a). (e) Schematic picture of the band structure of the MoSe_2_/CrSBr heterostructure. Dark and light colors correspond to filled and empty states, respectively. Process (1) indicates the quenching of the MoSe_2_ intralayer PL. Processes (2) and (3) visualize the charge-transfer process when pumping the CrSBr efficiently. All experiments are performed at *T* = 15 K.

Interestingly, on the heterostructure, we observe a strong pump–probe signal, even if we pump below the probe energy. For detailed analysis, we perform a series of DR experiments, where we keep the probe-energy window fixed at 1633 meV, and vary the pump windows as indicated by the green-shaded areas and arrows in Figure 3a. Figure 3d shows exemplary DR time traces for low (upper panel) and high pump energies (lower panel). A number of intriguing observations can be made: (i) Within the measurement window of 200 ps, no significant decay of the signal can be recognized (Figure 2d). The lifetimes are in the few-nanoseconds range, i.e., about 3 orders of magnitude longer than on the ML-MoSe_2_/SiO_2_ reference region (see Figure S2 of the SI), since we still observe a small nonzero signal at negative time delays, and the time between subsequent pulses is 12.5 ns in our laser system (for DR experiments with a longer time window, see Figure S6 of the SI). (ii) If we pump the CrSBr part efficiently, i.e., polarized parallel to the **b̂** axis, the instantaneous (within our time resolution) signal jump at zero time delay is stronger than for a pump polarization parallel to **â** (upper panel of Figure 3d). Furthermore, there is even a second, much slower built-up time of the signal of ∼43 ps (see red exponential fit line in Figure 3d) visible for pump polarization parallel to **b̂** in contrast to the perpendicular polarization, where the signal is almost time-independent within the measured time range. (iii) For high pump energy (lower panel of Figure 3d), the signal amplitudes at zero time delay are of equal height for both polarization directions, while the slower second built-up process for polarization parallel to **b̂** is still present. For systematic analysis, we plot in Figure 3b the amplitudes of the DR signals, |*R*_a_| and |*R*_b_|, as indicated in Figure 3d, versus the pump energy. The solid lines are fits of Lorentzians, centered at an energy of 1640 meV. In Figure 3c, the measured amplitudes are evaluated according to (*R*_b_ – *R*_a_)/(*R*_b_ + *R*_a_) to obtain the degree of optical anisotropy. At low pump energy, the anisotropy is at about 60%. If the pump energy approaches the MoSe_2_ transition at 1640 meV from below, the anisotropy goes down to a value of around 10%, and stays there for energies above the MoSe_2_ transition. This is contrary to measurements of the optical anisotropy of bulk CrSBr (position A) by linearly polarized PL-excitation experiments (see SI). In the energetic range of the MoSe_2_ transition, the anisotropy in bulk CrSBr is still above 80%.

At this point, the question of the origin of the very long-lived transient DR signals in Figure 3d arises. It clearly shows resonance behavior when approaching the MoSe_2_ optical transition (cf. Figure 3b). We exclude Pauli blocking by photoexcited electron–hole pairs (or excitons) in the MoSe_2_ layer as the origin of the long-lived DR signals by time-resolved PL experiments (Figure 2e). They deliver lifetimes of the broad heterostructure PL between about 11 and 6.5 ps. We believe that the long-lived DR signal is caused by a photoinduced charge transfer between the first CrSBr- and the MoSe_2_ layer, as schematically depicted in Figure 3e: Electrons, excited predominantly in the first CrSBr layer [process (2) in Figure 3e] tunnel into the MoSe_2_ valence band within a very short time, below our temporal resolution of about 0.2 ps, leading to an almost instantaneous decrease of the hole concentration. Due to the proximity interaction, this tunneling process is partially spin-allowed. The holes, left behind in the valence band of CrSBr, tunnel into the bulk due to the band bending. Therefore, reaching equilibrium again takes a long time, presumably in the ns range, as our experiments suggest. The slower, second built-up process can be explained by electrons, excited in deeper bulk layers [e.g., process (3) in Figure 3e], which have a lower tunneling probability since this process is spin-forbidden between antiferromagnetically oriented CrSBr bulk layers.

**Figure 4:**
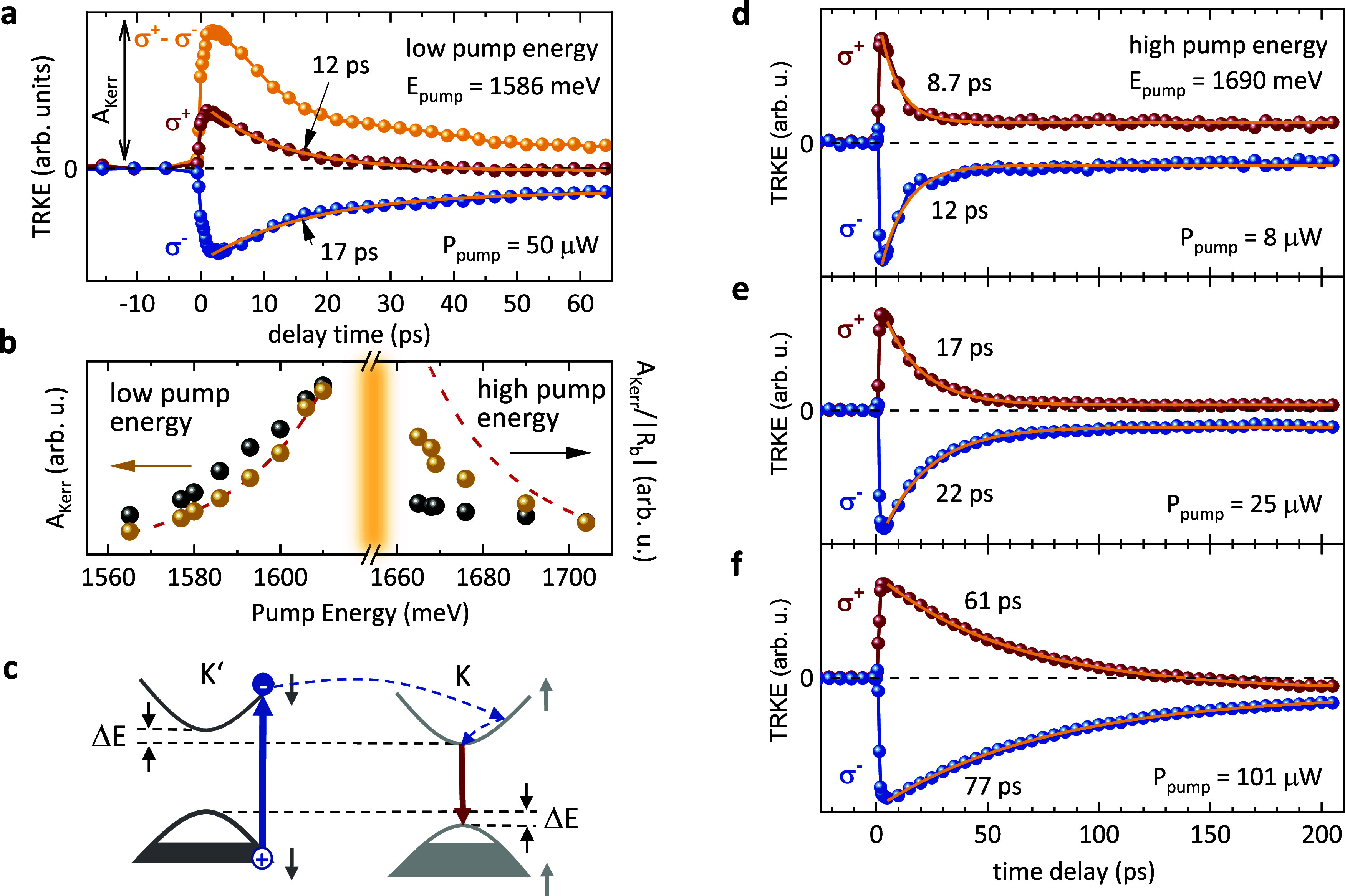
TRKE experiments on the ML-MoSe_2_/CrSBr heterostructure, spot B1. (a) TRKE traces of the ML-MoSe_2_/CrSBr heterostructure for a pump energy of 1586 meV, probe energy of 1633 meV, and different polarizations of the pump pulses, as indicated in the figure. (b) Kerr amplitudes, as defined in panel (a), versus pump energy (golden dots). The black dots are data points, normalized by the simultaneously measured RC amplitudes. (c) Schematic picture, visualizing the creation of a pump-induced spin polarization of background holes. (d–f) TRKE traces at high pump energy, centered at 1690 meV, for (d) low, (e) intermediate, and (f) high pump power. All experiments are performed at *T* = 15 K.

We continue by discussing the corresponding TRKE measurements. In Figure 4a, we compare TRKE traces for circularly polarized pump pulses of both helicities (red and blue dots), measured under conditions similar to those in the DR experiments, displayed in Figure 3d. From the TRKE traces, a helicity-independent background has been subtracted (see SI). We plot in Figure 4b the signal amplitudes of *A*_Kerr_, as defined in Figure 4a, versus pump energy (golden dots). The dashed red line is a fit with a Lorentzian, centered at 1640 meV. For low pump energies, this suggests a resonance with the transition at 1640 meV (the Fermi-edge transition). Since also the pump-induced number of transferred charges increases with increasing pump energy, we normalize *A*_Kerr_ by dividing it by the simultaneously measured amplitude of the DR signal |*R*_b_|. The resulting data points (black dots in Figure 4b) suggest that also the relative valley polarization increases when approaching the MoSe_2_ transition from below.

**Figure 5:**
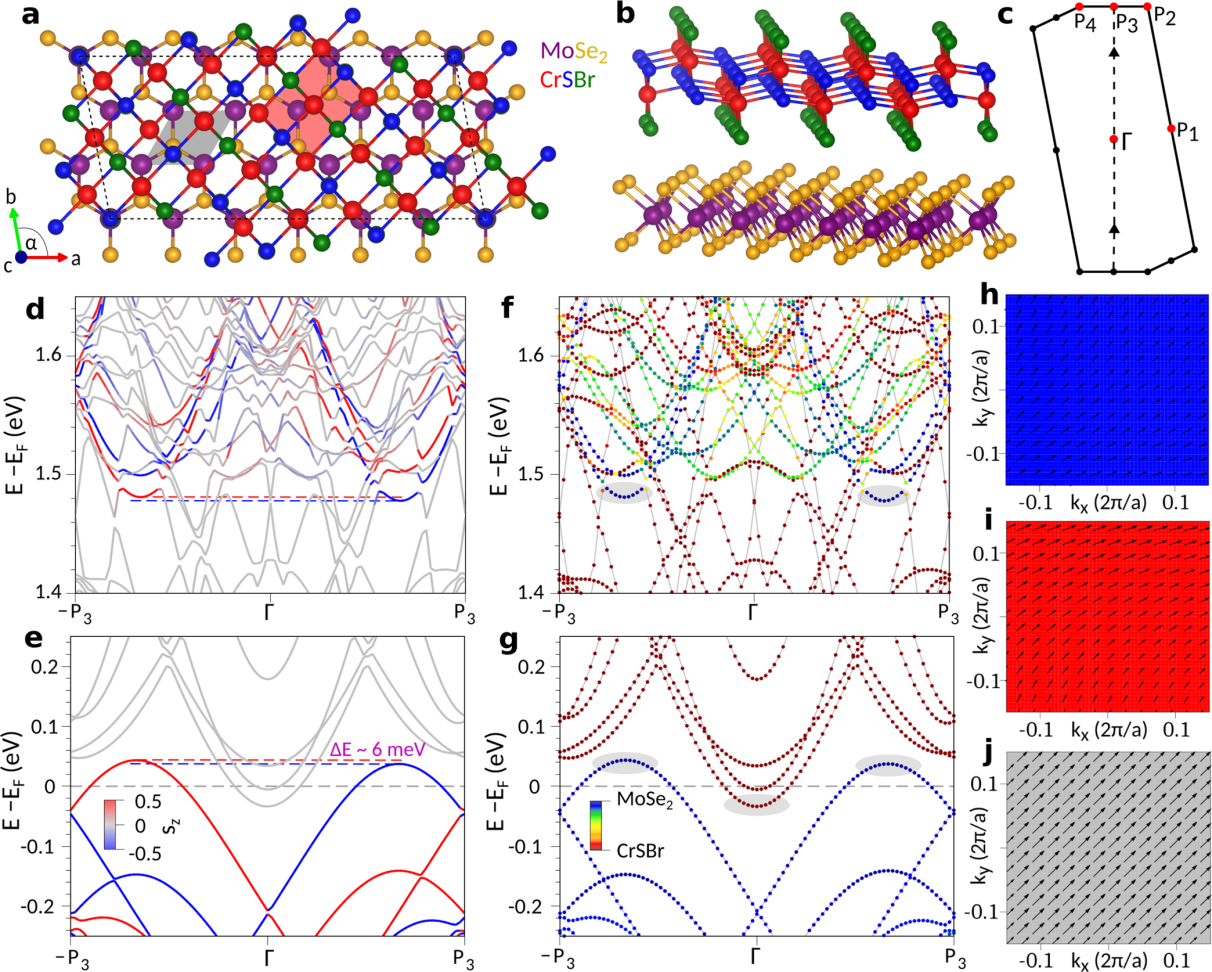
Geometry, Brillouin zone, and band structure of a MoSe_2_/CrSBr heterobilayer. (a, b) Top and side view of the MoSe_2_/CrSBr heterostructure. The gray (red) shaded area indicates the monolayer MoSe_2_ (CrSBr) unit cell, while the dashed line is the heterostructure unit cell. (c) The corresponding Brillouin zone, where we label high-symmetry points. The relevant MoSe_2_ valleys are folded along the path −P_3_–Γ–P_3_, indicated by the dashed line. (d, e) The calculated band structure near the band edges of MoSe_2_, including spin–orbit coupling and the in-plane magnetism of CrSBr. The color code represents the *s*_*z*_ spin expectation value. The dashed red/blue lines indicate the splitting of the valleys. (f, g) The same as (d, e), but the color code represents the projection onto the layers. The band edges are highlighted by gray-shaded areas. (h–j) Spin textures of the MoSe_2_ valence band edge valleys near P_3_ and −P_3_, and of the CrSBr conduction band edge valley near Γ. The color represents *s_z_*, while arrows correspond to *s*_*x*_ and *s*_*y*_.

When comparing the pump-energy dependencies of the CrSBr-related anisotropy (Figure 3c), and the TRKE amplitudes (Figure 4b), a contrasting behavior is obvious: By approaching the MoSe_2_ resonance, the optical anisotropy decreases strongly (Figure 3c), while the Kerr amplitude increases (Figure 4b). This can be explained by our band structure calculations of the heterostructure (see Figure 5, below): The states of the MoSe_2_-like conduction and valence bands are predominantly localized in the MoSe_2_ layer; i.e., they provide the helical selection rules. On the other hand, at energies below the MoSe_2_-like conduction band, the corresponding states are localized in the CrSBr layer, which imprint the CrSBr-related anisotropy onto the optical properties of the heterostructure.

The TRKE amplitude is a measure of the pump-induced valley polarization. Following from this, in nonmagnetic ML-TMDC samples, the TRKE traces with opposite pump helicities are symmetric with respect to the zero line (black dashed line in Figure 4a), due to time-reversal symmetry. Obviously, this is not the case for the TRKE traces in Figure 4a (red and blue dots), which are strongly asymmetric, pointing toward a violation of time-reversal symmetry. All TRKE traces in Figure 4a,d,e,f can be nicely fitted by monoexponential decays with constant background (solid yellow lines in Figure 4a,d,e,f). The constant background is due to a long-lived, pump-induced spin polarization of the resident holes, as it has been reported previously for background electrons and holes.^42−46^ A likely origin for the asymmetry could be the ground-state valley splitting between the K and K′ valleys due to a violation of time-reversal symmetry, as depicted in Figure 4c (and Figure 2a). Electrons, excited by σ^–^ pump photons, can scatter from the K′ valley to the K valley and recombine there with background holes of the Fermi sea. Because of the much smaller spin–orbit splitting of the conduction band, in comparison to the valence band,^32^ electrons have a higher probability of spinflip scattering than holes.^47^ The whole process increases the hole density in the K′ valley and reduces it in K. The displayed process in Figure 4c has a larger probability than the time-reversed one, i.e., when exciting the K valley by the opposite helicity, because of the larger phase space for scattering processes. Hence, the observed asymmetry can be taken as a further fingerprint of a ground-state valley splitting due to proximity-induced exchange interaction (see ref (48) for a similar asymmetry due to an external magnetic field).

We tested the pump-power dependence of the asymmetry for a fixed pump energy of 1690 meV (high pump energy) for three different pump powers in Figure 4d–f. The asymmetry increases from a low pump power (Figure 4d) to a high pump power (Figure 4f). Moreover, for σ^+^ versus σ^–^ pump helicity, we obtain consistently slightly different decay times of the TRKE traces (valley-polarization lifetimes). This also points toward an asymmetry between K and K′ valleys. For increasing pump power, the valley-polarization lifetimes increase up to a maximal value of 77 ps, i.e., more than 2 orders of magnitude longer than the valley lifetime of excitons in ML-MoSe_2_ (∼0.2 ps, see SI and ref (49)). We emphasize that we do not have evidence that the increase in valley lifetime is connected to the valley splitting. It may be likely that the prolongation is solely caused by the strong p-type doping of the MoSe_2_ layer, as a consequence of the type-III band alignment. In ref (47), e.g., a prolongation of valley lifetime at large electron/hole densities was reported for gated MoTe_2_ monolayers. Clearly, future experiments on gated MoSe_2_/CrSBr heterostructures are highly desired to disentangle pure carrier-density-related from proximity-induced influences on the valley lifetime. There are three main reasons for prolonged valley lifetimes in nonmagnetic TMDC monolayers with high carrier densities, which may be relevant for our case. First, for resident charge/spin carriers, the electron–hole exchange mechanism,^50^ which is responsible for the ultrafast valley relaxation of excitons in ML-MoSe_2_,^51−54^ is not applicable. Second, for valley relaxation of holes, hole spinflips are required.^47^ Those have a low probability for valence-band states in TMDCs because of the large spin–orbit splitting. Third, a spin polarization of resident carriers, as it can be reached by the optical pumping, stabilizes the spin polarization via the Hartree–Fock term of the particle–particle interaction:^55^ In experiments on n-doped GaAs quantum wells, it was shown that an initial spin polarization of ∼30% leads to an increase of the spin dephasing time by about an order of magnitude. Calculations with and without the Hartree–Fock term in the particle–particle interaction proved that the spin–spin interaction is the main stabilizing factor of the spin polarization.^55^

### First-Principles Calculations

For the calculations (for details, see Methods and SI), we consider a MoSe_2_/CrSBr heterobilayer, since the dominant effects of hybridization take place between the MoSe_2_ layer and the first CrSBr layer. Figure 5a,5b shows top and side views of the assumed heterobilayer with 114 atoms in the supercell. The monolayer MoSe_2_ and CrSBr unit cells are indicated by the gray- and red-shaded areas, respectively. The heterobilayer supercell is outlined by black dashed lines. The resulting Brillouin zone is shown in Figure 5c, where we folded the relevant MoSe_2_ valleys along the path −P_3_–Γ–P_3_, which is indicated by a dashed line. The solid triangles mark the positions of the K and K′ valleys of ML-MoSe_2_. To get a realistic description of the heterostructure dispersion, we consider noncollinear magnetism and spin–orbit coupling in the calculations.

The resulting band structure of the heterobilayer is displayed in Figure 5d–5g. The two upper panels (Figure 5d,f) show the region around the conduction band of MoSe_2_, while a zoom into the valence-band region is shown in the two lower panels (Figure 5e,g). The spin directions of the bands are highlighted in Figure 5d,e by the indicated color code. One can see that the out-of-plane spin directions of ML-MoSe_2_ at the K valleys are largely conserved in the heterobilayer, which was previously found also for other systems.^56−58^ We find the valence-band edges of MoSe_2_ for the two valleys K and K′ (in our case folded toward P_3_ and −P_3_) about 37.4 and 43.6 meV above the Fermi level, resulting in a splitting of ∼6 meV (see Figure 5e). This nicely confirms our schematic picture in Figure 2a, and is in fair agreement with the experimentally observed splitting of ∼1.6 meV. The lifting of the valley degeneracy in the heterobilayer can be understood in the following way: Due to spin–orbit coupling, the magnetization induced in the MoSe_2_ layer from the in-plane-magnetized CrSBr acquires an out-of-plane component; see Table I of the SI. The magnetization of the stack becomes noncollinear. Similar to the effect of an external magnetic field along *z*, this induced out-of-plane magnetization lifts the valley degeneracy, of about 6 meV. The CrSBr conduction-band edge is ∼33.6 meV below the Fermi level. The hole densities from K and K′ valleys of MoSe_2_ are *p* = 5.46 × 10^12^ cm^–2^ and *p*′ = 4.69 × 10^12^ cm^–2^ correspondingly.

In Figure 5f,g, the projection of the bands onto the layers is color coded. Relevant band edges are highlighted by gray-shaded areas. We can see that, indeed, the band extrema, reminiscent of the conduction-band minima and valence-band maxima of ML-MoSe_2_, are located in the MoSe_2_ layer, while the bands in between are dominantly in the CrSBr layer. Figure 5h–j show the calculated spin textures of the MoSe_2_ K/K′- and the CrSBr Γ band edges near the Fermi level. The band edges in MoSe_2_ and CrSBr that contribute to charge transfer retain their spin character to a large extent but also acquire a proximitized character due to the vdW interface.

## Conclusions

We have investigated a vdW heterostructure consisting of ML-MoSe_2_ and the antiferromagnetic vdW crystal CrSBr. The following findings have been made: (i) identification of a very long-lived dynamic charge-transfer process in the heterostructure due to a type-III band alignment, alongside strong p-type doping of the MoSe_2_. (ii) Both, from the experimental as well as theoretical side, we have found evidence for a breaking of time-reversal symmetry, due to a proximity-induced exchange interaction. Experimentally, we estimate the proximity-induced spin splitting to be about 1.6 meV. (iii) We have detected a valley lifetime of spin-polarized charge carriers in the MoSe_2_ layer, which is more than 2 orders of magnitude longer than for plain MoSe_2_. Overall, our investigations exemplify the optimization of the optical properties of ML-MoSe_2_ by vdW engineering.

## Methods

### Sample Preparation

The investigated heterostructure has been prepared using a dry transfer technique.^59^ It consists of an ∼35 nm-thick CrSBr layer, which corresponds to approximately 44 layers, and a monolayer of MoSe_2_ on top. The whole sample is capped by a ∼20 nm-thick layer of hexagonal boron nitride (hBN). The used flakes, i.e., monolayer MoSe_2_, bulk CrSBr, and hBN, were obtained by mechanical exfoliation on SiO_2_ substrates. For preparation of the layer stack, at first, the hBN layer is picked up by a polycarbonate (PC) film at 70 °C. Then, the hBN is used to pick up the monolayer MoSe_2_ and CrSBr flakes successively. The hBN-MoSe_2_–CrSBr stack is then released on a clean SiO_2_ substrate with Au markers by melting the PC film at temperatures around 180 °C. Afterward, the PC film, covering the stack, is removed from the surface by dissolving it in chloroform. Finally, the sample is annealed in Ar atmosphere at 150 °C for 4 h. The height profile of the heterostructure is measured by atomic-force microscopy.

The CrSBr source crystals were grown by chemical vapor transport, as reported in ref (60). For characterization, powder X-ray diffraction, Raman and infrared spectroscopy, energy-dispersive X-ray analysis (EDX), high-resolution transmission-electron microscopy, and SQUID magnetometry are applied. The MoSe_2_ source crystal was purchased from SPI Supplies.

### Optical Experiments

#### Pump–Probe Experiments

The pump–probe experiments are performed with a tunable two-color pump–probe setup. The pulse train of a mode-locked Ti:sapphire laser (Spectra Physics Tsunami, pulse duration ∼100 fs) is divided via a beam splitter into two beams, which can be delayed with respect to each other by a mechanical delay line. One of the beams (probe beam) is focused into an optical fiber to produce a white-light continuum. Afterward, specific spectral probe regions are cut out of the continuum via high-quality edge filters. Motorized rotation of the filters allows tuning of the spectral edges continuously. The pump beam is shaped by edge filters, only. Both beams are focused via a 60× microscope objective onto the sample to a spot diameter of ∼2 μm, where the beams overlap. The sample is mounted on the coldfinger of a He-flow cryostat on top of a Neodymium permanent magnet, which provides a small canting field on the sample, between about 120 and 190 mT. However, this small magnetic field has no significant influence on our reported experiments (see experiments without the Neodymium magnet and with an external magnetic field in the SI). A motorized *x*-*y* stage allows for scanning experiments. Different detection techniques are available. There is an optical bridge with two photodiodes, where either the Kerr rotation or ellipticity of the probe beam after reflection on the sample can be determined by the difference signal of the two photodiodes using lockin technique. For time-resolved Kerr ellipticity (TRKE), a circularly polarized pump pulse excites the sample. After an adjustable time delay, the linearly polarized probe beam is reflected on the sample. If a nonzero magnetization is present in the sample, e.g., due to a valley population difference in the MoSe_2_ layer caused by the absorption of circularly polarized pump photons, the polarization of the probe beam acquires an ellipticity. The ellipticity is proportional to the difference α_+_–α_–_ in absorption coefficients α_±_ for σ^+^- and σ^–^-polarized light. For ML-MoSe_2_, α_+_–α_–_ is nonzero for unequal populations of the K and K′ valleys. Hence, the TRKE amplitude is a measure of the pump-induced valley polarization. Simultaneously, the total probe intensity is measured by the photodiode sum signal for transient differential-reflectivity (DR) experiments. Alternatively, the reflected signal can be sent into a grating spectrometer with a CCD detector for PL or RC measurements. This means that we can perform, e.g., four different experiments on the same sample spot, in the same measurement run: PL, RC, TRKE, and DR. Via TRKE, we measure the temporal dynamics of the valley or spin polarization and, via DR, the exciton or carrier lifetimes.

#### Time-Resolved Photoluminescence

For time-resolved PL experiments, a microscope setup with coldfinger cryostat, 100× microscope objective, grating spectrometer, and a streak camera as detector (Hamamatsu synchroscan) is used. The PL is excited by a mode-locked Ti:sapphire laser with 100 fs pulses.

For more details and a sketch of the experimental setup, see SI.

### DFT Calculations

#### Structural Setup

The MoSe_2_/CrSBr heterostructure was setup with the atomic simulation environment (ASE)(62) and the CellMatch code,^63^ implementing the coincidence lattice method.^64,65^ The lattice constants of CrSBr within the heterostructure are *a* = 3.557 Å and *b* = 4.742 Å, in close agreement with experimental values,^66,67^ while the MoSe_2_ layer is kept unstrained with a lattice constant of 3.288 Å.^68^ Therefore, the individual monolayers are barely strained in our heterostructure, and we should be able to reliably extract band offsets as well as proximity exchange effects. In order to simulate quasi-two-dimensional (2D) systems, we add a vacuum of about 18 Å to avoid interactions between periodic images in our slab geometry. The resulting supercell has 114 atoms, with the lattice parameters of |*a*| = 19.728 Å, |*b*| = 8.699 Å, |*c*| = 30.219 Å, and α = 100.893°. The relaxed average interlayer distance between the layers is *d* = 3.420 Å. For more details, see SI.

#### Computational Details

The electronic structure calculations and structural relaxations of the MoSe_2_/CrSBr heterostructure are performed by DFT^69^ with Quantum ESPRESSO.^70^ Self-consistent calculations are carried out with a *k*-point sampling of 12 × 18 × 1. We performed open-shell calculations that provided the spin-polarized ground state of the CrSBr monolayer. A Hubbard parameter of *U* = 2.0 eV is used for Cr d-orbitals. We use an energy cutoff for charge density of 560 Ry and the kinetic energy cutoff for wave functions is 70 Ry for the relativistic pseudopotentials with the projector augmented wave method^71^ with the Perdew–Burke–Ernzerhof exchange-correlation functional.^72^ For the relaxation of the heterostructures, we add DFT-D2 vdW corrections^73−75^ and use quasi-Newton algorithm based on the trust radius procedure. To get proper interlayer distances and to capture possible moiré reconstructions, we allow all atoms to move freely within the heterostructure geometry during relaxation. Relaxation is performed until every component of each force is reduced below 2 × 10^–4^ [Ry/*a*_0_], where *a*_0_ is the Bohr radius. For more details, see Supporting Information.

## Supporting Information Available

Supplementary experimental details and DFT calculations on MoSe_2_/CrSBr heterobilayer (PDF)

## Notes

A.B., K.Z., C.S.d.B., P.E.F.J., T.S.G., J.I.-A., S.M.-V., C.B.-C., K.W., T.T., J.F., H.S.J.v.d.Z., Y.G.G., and C.S. Proximity-induced exchange interaction and prolonged valley lifetime in MoSe2/CrSBr van-der-Waals heterostructure with orthogonal spin–orbit fields. 2024, Research Square. DOI: 10.21203/rs.3.rs-4324718/v1 (accessed 10/05/2024).
