# Supplementary material for: Proximity-Induced Exchange Interaction and Prolonged Valley Lifetime in MoSe2/CrSBr Van-Der-Waals Heterostructure with Orthogonal Spin Textures
Source: ACS Nano. 2024 Oct 28;18(45):31044–54. doi: 10.1021/acsnano.4c07336 (PMC11562783; doi:10.1021/acsnano.4c07336)
Supplement: Supplementary file 1 — nn4c07336_si_001.pdf [file nn4c07336_si_001.pdf]

Supplementary Information for:
Proximity-induced exchange interaction and prolonged valley lifetime in $MoSe_2$/CrSBr van-der-Waals heterostructure with orthogonal spin textures

Andreas Beer,[1] Klaus Zollner,[2] Caique Serati de Brito,[1,3] Paulo E. Faria Junior,[2] Philipp Parzefall,[1] Talieh S. Ghiasi,[4] Josep Ingla-Aynés,[4] Samuel Mañas-Valero,[4] Carla Boix-Constant,[5] Kenji Watanabe,[6] Takashi Taniguchi,[6] Jaroslav Fabian,[2] Herre S. J. van der Zant,[4] Yara Galvão Gobato,[3] and Christian Schüller[1,*]

[1]*Institut für Experimentelle und Angewandte Physik, Universität Regensburg, D-93040 Regensburg, Germany*

[2]*Institute of Theoretical Physics, University of Regensburg, 93040 Regensburg, Germany*

[3]*Physics Department, Federal University of São Carlos, São Carlos, SP, 13565-905, Brazil*

[4]*Kavli Institute of Nanoscience, Delft University of Technology, Lorentzweg 1, 2628 CJ Delft, The Netherlands*

[5]*Instituto de Ciencia Molecular (ICMol), Universitat de València, Catedrático José Beltrán 2, Paterna 46980, Spain*

[6]*Research Center for Materials Nanoarchitectonics, National Institute for Materials Science, 1-1 Namiki, Tsukuba 305-0044, Japan*

(Dated: October 11, 2024)

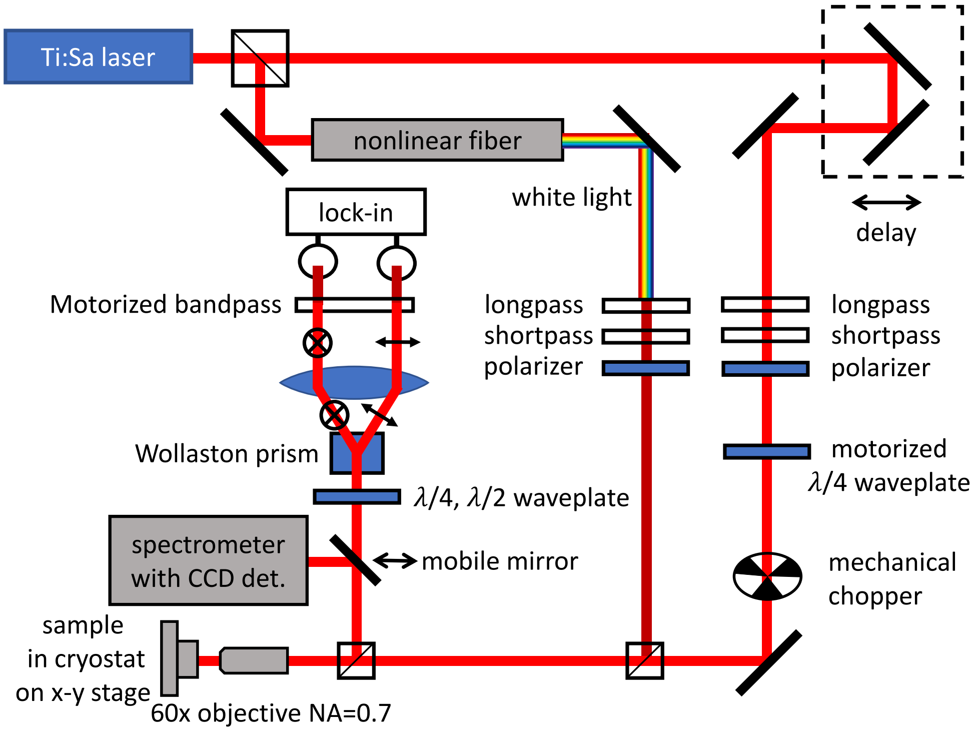

FIG. S1. — **Experimental Setup.** Sketch of the experimental setup, used for tunable two-color pump-probe experiments, PL and RC experiments.

SUPPLEMENTARY EXPERIMENTAL DETAILS

Experimental Setup

Photoluminescence (PL), reflectance contrast (RC), and time-resolved pump-probe experiments are performed with a tunable two-color pump-probe setup. Figure S1 shows a schematic picture of the used setup. The pulse train of a mode-locked Ti:Sapphire laser (Spectra Physics Tsunami, pulse duration $\sim$ 100 fs) is divided via a beam splitter into two beams, which can be delayed with respect to each other by a mechanical delay line. One of the beams (probe beam) is focused into an optical nonlinear fiber to produce a white-light continuum. Shortpass and longpass optical filters (Semrock VersaChrome Edge tunable shortpass and longpass) in the probe and the pump beam ensure that both beams can be spectrally separated for detection. Both beams are focused co-linearly onto the sample by a 60x microscope objective (NA=0.7) to a spot diameter of ~ 2 μm. The sample is mounted

* christian.schueller@ur.de

on the cold finger of a He-flow cryostat, on top of a Neodymium permanent magnet, which provides a small canting field on the sample, between about 120 mT and 190 mT. However, this small magnetic field has no measurable influence on our reported experiments. The He-flow cryostat is sitting on a motorized x-y stage for automated positioning. After polarization selection (Wollaston prism) a motorized bandpass filter (Semrock VersaChrome Edge tunable shortpass and longpass) is used to select a specific energy window for the probe pulses. Motorized rotation of the filters allows to tune the spectral edges continuously and with repeated accuracy. Different detection techniques are available. There is an optical bridge with two photodiodes, where either the Kerr rotation, by using a $\lambda/2$ plate, or ellipticity, by using a $\lambda/4$ plate, of the probe beam after reflection on the sample can be determined by the difference signal of the two photodiodes. For detection, a lockin-amplifier (Stanford Instruments) is used, with reference signal from the mechanically-chopped pump beam. Simultaneously to the difference signal, the total probe intensity is measured by the photodiode sum signal for transient differential-reflectivity experiments. Alternatively, the reflected signal can be sent via a moveable mirror into a grating spectrometer with CCD detector for PL or RC measurements. For RC measurements, the spectrally-broad pump pulses are used. The RC spectra are determined via $(R_S - R_R)/(R_R - R_D)$, where R_S, R_R and R_D are the reflectance spectra of the laser pulses on the sample, on a reference region and a dark spectrum, respectively. This means, we can perform, e.g., four different experiments on the same sample spot, in the same measurement run: PL, RC, time-resolved Kerr ellipticity (TRKE), and transient differential reflectivity (DR). Via TRKE, we measure the temporal dynamics of the valley or spin polarization, and, via DR, the exciton or carrier lifetimes.

For time-resolved PL experiments, another microscope setup with cold-finger cryostat and a streak camera as detector (Hamamatsu synchroscan) is used. For excitation, a mode-locked Ti:Sapphire laser (Spectra Physics Tsunami) with 100 fs pulses is used. The PL is dispersed in a grating spectrometer with 30 cm focal length. The spectrometer has two output ports, where a CCD camera (Orca) for time-integrated PL, and the streak camera (Hamamatsu) for time-resolved PL are mounted. The DOCP measurements in the manuscript and below are recorded with the Orca CCD.

Pump-Probe Experiments on $MoSe_2/SiO_2$ Reference Region

Figure S2 shows a set of experiments on the $MoSe_2$ reference region (position C). $MoSe_2$ on SiO_2 is known to be slightly n-doped, i.e., the trions, T, are negatively-charged excitons. Figures S2b and S2c display TRKE and DR time traces, respectively. The pump pulses excite the sample energetically above the excitonic transitions. The probe pulses are either probing the neutral exciton X, or the trion T. The spectral regions of the pump/probe pulses are indicated in Fig. S2a, relative to the excitonic PL lines and the RC spectrum. For the TRKE experiments, we plot the difference of traces, excited with σ^+- ($TRKE^+$) and σ^--polarized pump pulses ($TRKE^-$), to eliminate helicity-insensitive background contributions [1]. Decay times, as extracted by mono-exponential fits are given next to the curves in Figs. S2b and S2c. They confirm the well-known short valley- (Fig. S2b) [2] and exciton lifetimes (Fig. S2c) [3] of ML-$MoSe_2$, being in the sub-ps- and few-ps range, respectively. Actually, the decay time τ_D of a TRKE trace is determined by both, the valley-relaxation time τ_V and the exciton (or trion) lifetime τ_E via $1/\tau_D = 1/\tau_V + 1/\tau_E$, since also sole recombination of excitons (or trions) leads to a signal decay, even without any valley relaxation. Comparing numbers in Figs. S2b and S2c, we see that for X (red traces), $\tau_E >> \tau_D$, i.e., $\tau_V \sim \tau_D = 0.2$ ps. The very short τ_V of ML-$MoSe_2$ goes hand in hand with a very low DOCP in continuous-wave PL experiments (e.g., Ref. 4). For T (blue traces in Figs. S2b and S2c), the decay times of TRKE and DR are comparable, i.e., we have to compute the valley-relaxation time with the above relation, which gives $\tau_V \sim 1.4$ ps, which is significantly longer than for X. This can also be understood and is known from literature. For X, the electron-hole exchange mechanism [5–9] is dominant for valley relaxation, while for T the spinflip of a hole is required, which is much more inefficient [10]. As a side note, we can even resolve a time shift of ~ 0.4 ps in Figs. 2b and 2c, which it takes for X to capture an electron and transform into T in our experiment.

Optical anisotropy of bulk CrSBr

In Fig. S3 we investigate the optical anisotropy of bulk CrSBr in the energy range, relevant for the pump-probe experiments in the manuscript. Figure S3a displays the PL spectrum of the bulk CrSBr reference region (position A). The arrow marks the PL peak, which is

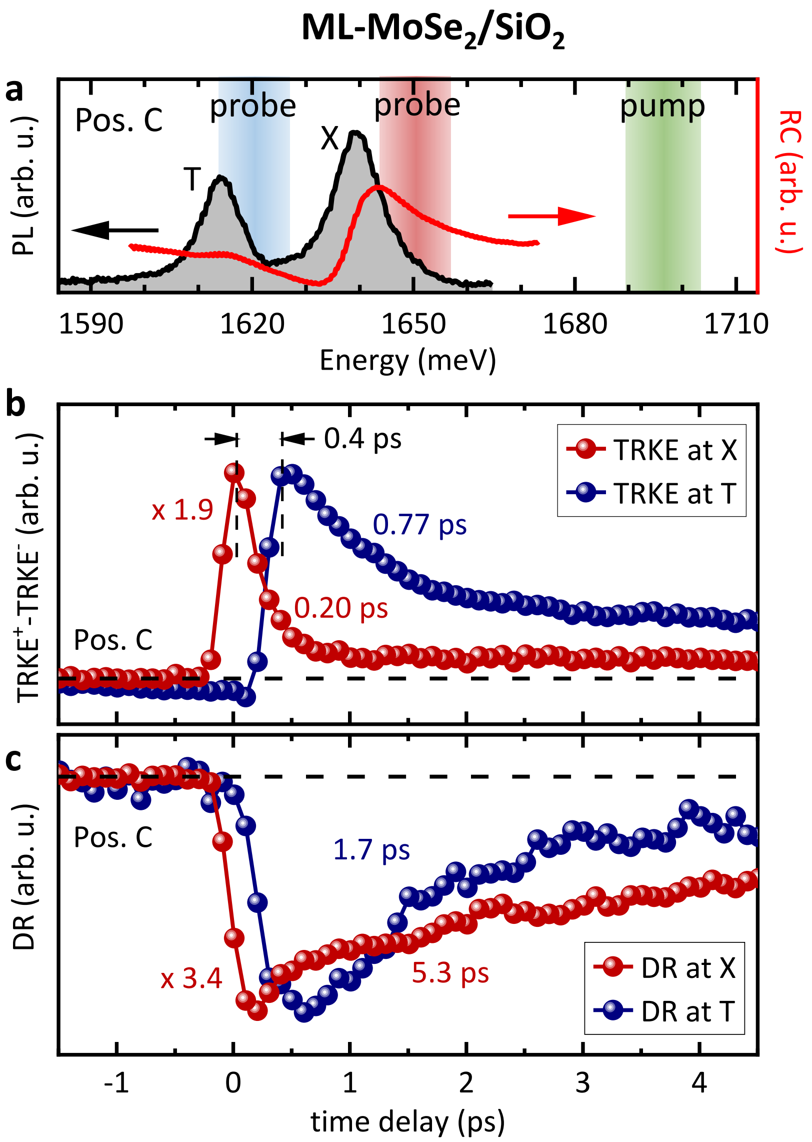

FIG. S2. — **Two-color pump-probe experiments on ML-MoSe$_2$/SiO$_2$, spot C.** (a) PL spectrum, taken at position C. The energy windows of the pump- and probe pulses, used in the TRKE and DR experiments, are highlighted by color-shaded areas. (b) TRKE time traces using circularly-polarized pump pulses, probing the valley polarization of the exciton (red dots) and the trion (blue dots). Plotted is the difference between measurements with σ^+ and σ^- pump pulses (TRKE$^+$-TRKE$^-$) to eliminate helicity-insensitive contributions. (c) Transient DR traces under the same experimental conditions as in (b) but with linearly-polarized pump- and probe pulses, probing the exciton and trion lifetimes.

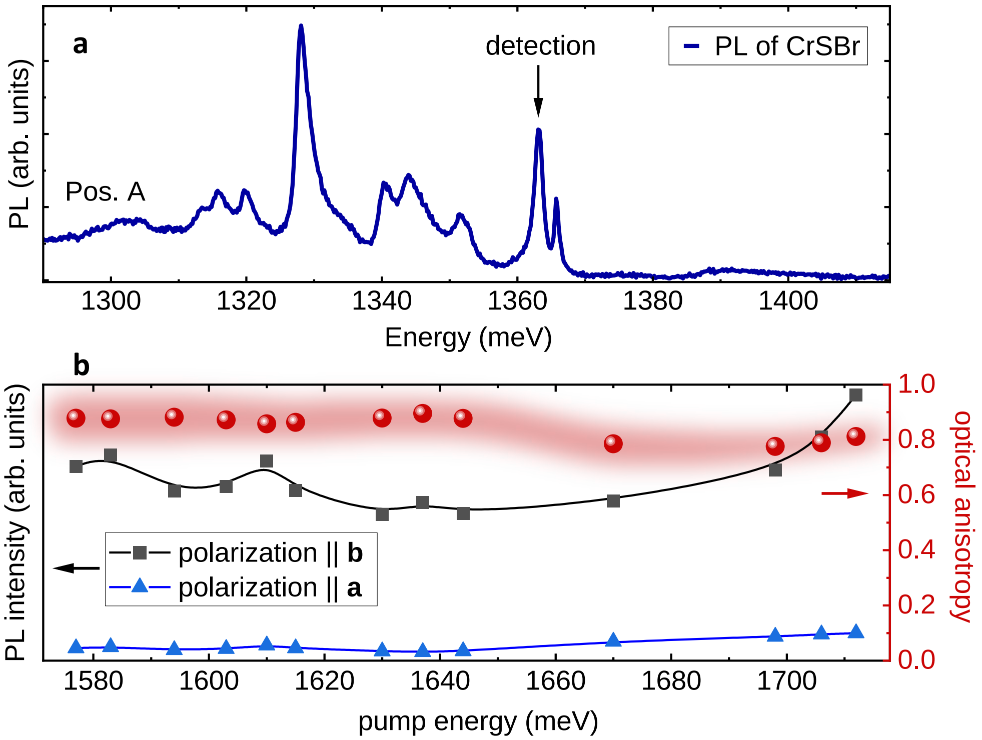

FIG. S3. — **Optical Anisotropy of CrSBr.** (a) PL spectrum of the bulk CrSBr reference region (position A) (b) PL intensity of the PL peak at 1363 meV versus pump-laser energy. The red dots show the degree of anisotropy of the emitted PL with respect to the direction of linear polarization of the excitation laser.

used for photoluminescence-excitation (PLE) detection. The recorded PLE data points are shown in Fig. S3b. For the black solid squares, the linear polarization of the excitation laser is parallel to the crystallographic $\hat{\mathbf{b}}$ axis (the magnetic easy axis), while it is parallel to $\hat{\mathbf{a}}$ (the magnetic intermediate axis) for the blue solid triangles. We compute the degree of optical anisotropy from the PLE data points by the relation $(I_b - I_a)/(I_b + I_a)$, where I_b (I_a) corresponds to the PL intensity with excitation polarization parallel to $\hat{\mathbf{b}}$ ($\hat{\mathbf{a}}$). One can see that in the region of 1620 meV to 1640 meV, i.e., the optical bandgap of the ML-$MoSe_2$/CrSBr heterostructure at position B1 with optimal contact, the optical anisotropy of bulk CrSBr is $> 80\%$. This contrasts with the results from the $MoSe_2$/CrSBr heterostructure (Fig. 3c of the manuscript), where the anisotropy goes down to about 10% in this spectral range.

Background substraction for TRKE experiments

In this section, the background-substraction procedure for TRKE measurements is described. As an example, we plot in Fig. S4a raw data, as recorded on position B1 (heterostructure region with optimal contact) for pump and probe energies, centered at 1678 meV and 1633 meV, respectively. The blue dots are traces, recorded with circularly-polarized pump pulses, as indicated in the figure. There is a constant, positive lockin signal present at negative time delays. This is unusual, since we use lockin detection with modulation of the pump beam by a mechanical chopper wheel (see above). Therefore, at negative time delay, i.e., before the pump pulse arrives at the sample, the signal should be zero. At positive time delays we expect to see the pump-induced changes to the system. To investigate the origin of the unusual signal at negative time delay, we block the probe beam and record a trace with pump beam, only (red dots in Fig. S4a). We get a time-independent signal (as expected, since the probe beam is blocked) but at finite positive value, which is exactly the same as the negative-time signal of the full measurements. Therefore, this background signal has to come from some unwanted stray light of the pump beam, which reaches the photodiodes of our optical-bridge detector (see setup above). Since the pump beam is modulated, the stray light is detected by the lockin amplifier. This can be due to imperfections of our used filters, which we use to separate pump from probe beams, and/or from unwanted reflections on, e.g., the filter surfaces. We, therefore, substract the trace, recorded with blocked probe beam from both traces, recorded with circularly-polarized pump beams. The background-corrected TRKE traces are displayed in Fig. S4b. If not otherwise stated, similar procedures are applied to all TRKE measurements, shown in the manuscript.

Coherent magnons in bulk CrSBr, sample position A

For completeness, we briefly discuss in the following TRKE experiments, which are conducted on the bulk CrSBr reference region (position A) of the sample. Interestingly, we observe in TRKE experiments on CrSBr with linearly-polarized pump pulses and approximately the same pump/probe energies as in the ML-$MoSe_2$ experiments, i.e., far above the optical bandgap of CrSBr, coherent oscillations with 25 GHz frequency (Fig. S5). In Fig. S5, a non-oscillating background signal has been substracted. Similar oscillations have been re-

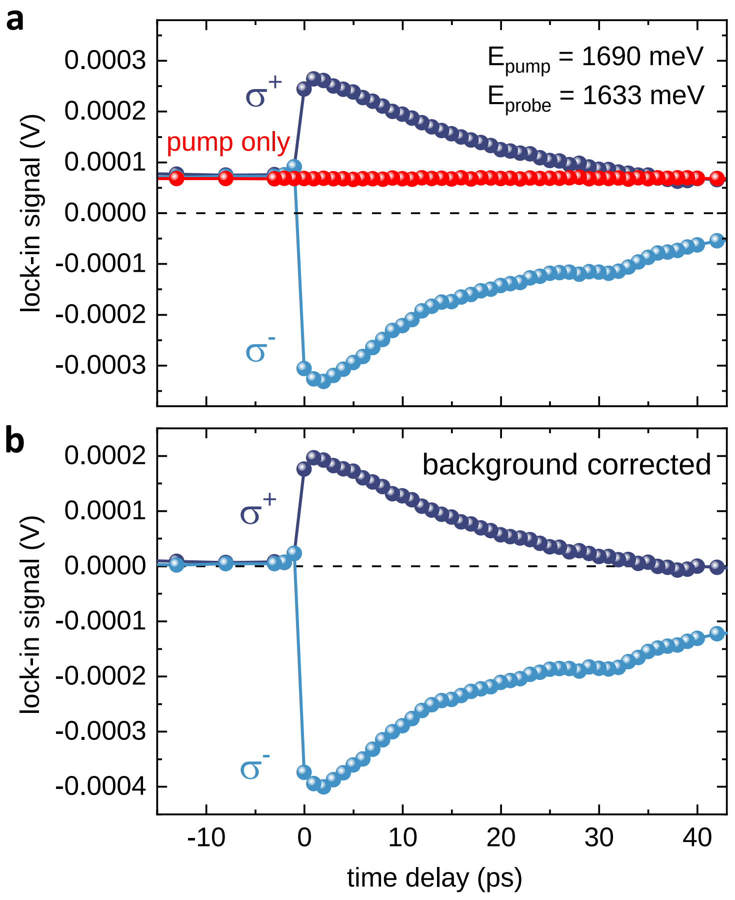

FIG. S4. — **Background Substraction for TRKE.** (a) TRKE traces, recorded on position B1 ($MoSe_2$/CrSBr heterostructure), with circularly-polarized pump pulses (blue dots), and with blocked probe beam (red dots). Pump and probe energy windows are centered at 1678 meV and 1633 meV, respectively. (b) TRKE traces after background correction, as described in the text.

ported recently in Refs. 11 and 12, and were interpret to result from thermal excitation of coherent in-plane magnons by the pump pulses. However, in Refs. 11 and 12, the CrSBr excitonic transition around 1360 meV was probed, and an exciton-magnon coupling was anticipated, while in our experiment we probe at an energy far above the optical bandgap. We note that in our TRKE experiments, displayed in Fig. S5, the pump/probe powers are about one order of magnitude larger than in all other experiments on the ML-$MoSe_2$- or the heterostructure regions. Furthermore, the signal strength in Fig. S5 is about one order of magnitude smaller than in the other experiments. Therefore, on the scale of the above presented TRKE experiments, the magnon oscillations would be at or below the noise level. The two traces in Fig. S5 for linear pump polarizations parallel to $\hat{\mathbf{a}}$ and $\hat{\mathbf{b}}$ show that the

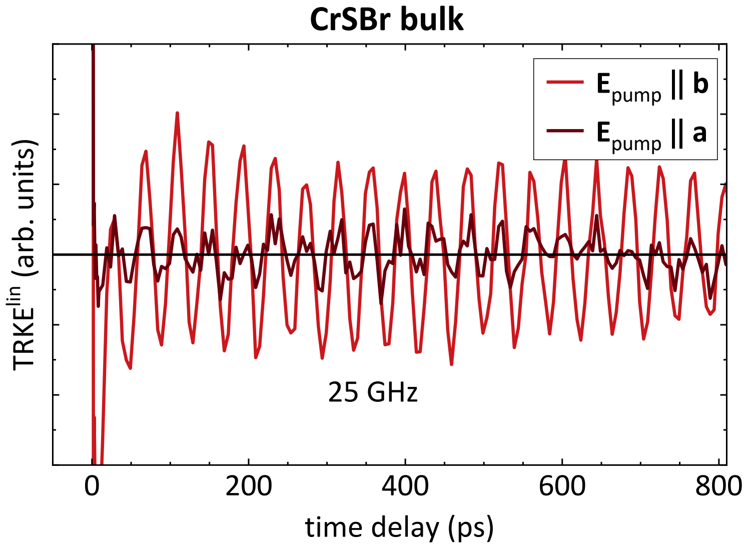

FIG. S5. — **TRKE experiments on bulk CrSBr, spot A.** TRKE traces of bulk CrSBr, taken with linearly-polarized pump pulses, where the polarization was either parallel to the $\hat{\mathbf{a}}$ or $\hat{\mathbf{b}}$ crystallographic axes of CrSBr. A non-oscillating background signal has been substracted. Pump- and probe energies were at 1697 meV and 1650 meV, respectively.

strong in-plane anisotropy of bulk CrSBr holds up to more than 200 meV excess energy above its optical bandgap, i.e., at the energy of the $MoSe_2$ optical bandgap. As shown in the manuscript, this is strongly different for the ML-$MoSe_2$/CrSBr heterostructure due to hybridization.

Differential reflectivity experiments for longer delay times, sample position B1

Figure S6 shows DR experiments, taken at sample position B1 of the ML-$MoSe_2$/CrSBr heterostructure for linear polarization directions of the pump pulses, being either parallel to the $\hat{\mathbf{a}}$ or $\hat{\mathbf{b}}$ axes of CrSBr. For $\mathbf{E_{pump}} \parallel \hat{\mathbf{a}}$, the DR signal is almost constant over the entire time range of 865 ps. For $\mathbf{E_{pump}} \parallel \hat{\mathbf{b}}$, after the steep initial jump of the signal at zero time delay, a slower second built-up, with a following decay, can be recognized. The central energies of pump and probe pulses are given in the figure. Both curves, together with a nonzero signal strength at negative time delay, indicate a lifetime of the DR signal in the nanosecond range, as discussed in the main manuscript.

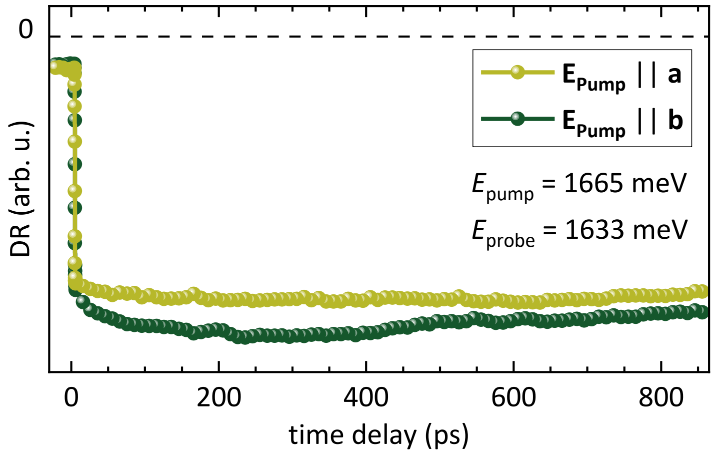

FIG. S6. — **DR experiments on ML-$MoSe_2$/CrSBr heterostructure, spot B1.** DR time traces for longer time delays up to 865 ps. The linear polarization directions of the pump pulses, as well as the central energies of pump and probe pulses are given in the figure.

Photoluminescence experiments with out-of-plane B field, sample position B1

Figure S7 shows the energetic positions of PL spectra for co-circularly-polarized excitation and detection for sample position B1. For excitation, a 1880 meV cw laser is used. The experiments are recorded in a magnet cryostat [helium closed-cycle cryostat with superconducting magnet coils (Attocube - Attodry1000) with magnetic fields up to 9 T, applied perpendicular to the heterostructure], without a Neodymium permanent magnet, i.e., without a canting field. An energetic splitting of $\sim$ 1.4 meV between both helicities at zero magnetic field, reminiscent of the observed splitting in the main body of the manuscript, can be recognized. From this, we can conclude that the canting field, present in the experiments of the main manuscript, does not have a significant influence on the results. Overall, the magnetic-field behavior is complex. One may conclude that an external magnetic field in the range < -6 T may compensate the proximity-induced zero-field splitting. However, for out-of-plane fields, oriented parallel to the magnetic hard axis of CrSBr, $\mathbf{B} \parallel \hat{\mathbf{c}}$, the antiferromagnet undergoes a forced phase transition into a ferromagnetic state at $|B| = 2.25$ T [32], indicated by vertical gray lines and small arrows in Fig. S7. At the phase transition, the band alignment in the heterostructure changes [32], which may also change the doping level in $MoSe_2$. This requires more detailed experimental and theoretical investigations, which is

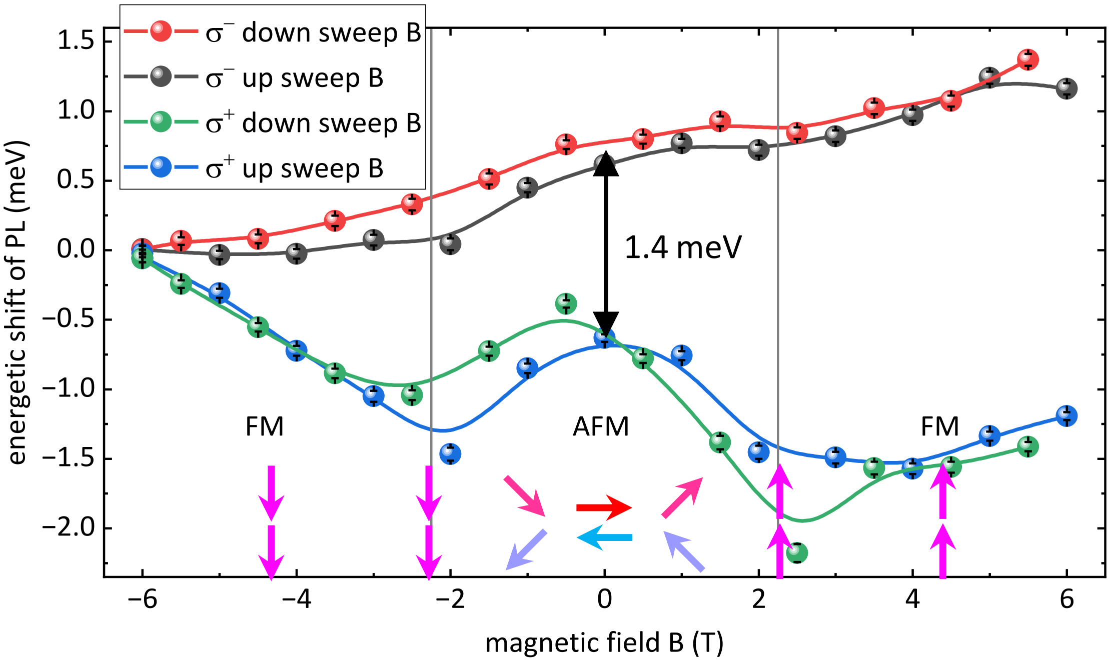

FIG. S7. — **PL experiments on ML-MoSe$_2$/CrSBr heterostructure with out-of-plane magnetic field, spot B1.** Energetic shifts of PL peak positions, given relative to the position for σ^- polarization at $B = 0$. Data for up- and down sweeps of the magnetic field are shown. There are small hysteresis effects observable. The solid lines are guides to the eye. Colored arrows account for magnetization in CrSBr.

work in progress.

DFT CALCULATIONS ON MOSE$_2$/CRSBR HETEROBILAYER

Structural Setup

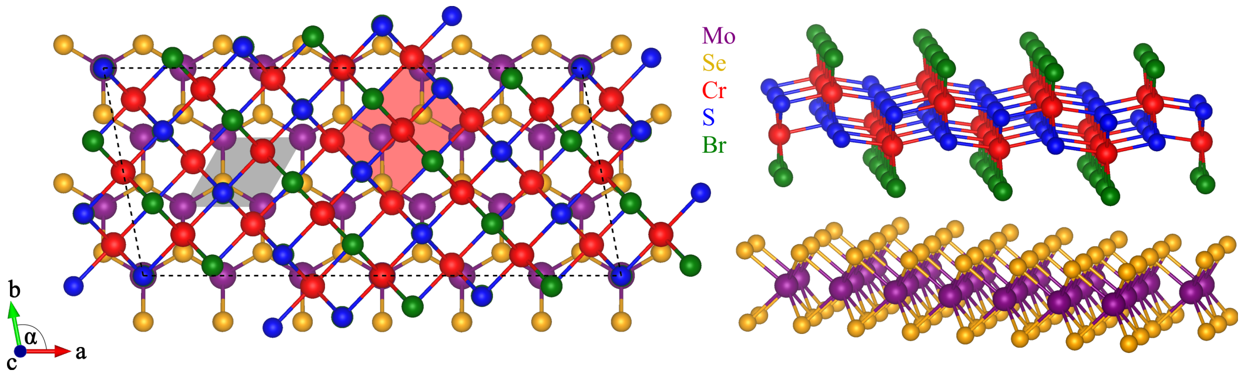

FIG. S8. — **Top and side view of the MoSe$_2$/CrSBr heterostructure.** The supercell has 114 atoms, with the lattice parameters of $|a| = 19.728$ Å, $|b| = 8.699$ Å, $|c| = 30.219$ Å, $\alpha = 100.893°$. The relaxed average interlayer distance between the layers is $d = 3.420$ Å. The grey (red) shaded area indicates the monolayer MoSe$_2$ (CrSBr) unit cell, while the dashed line is the heterostructure unit cell.

The MoSe$_2$/CrSBr heterostructure was set-up with the `atomic simulation environment (ASE)` [13] and the `CellMatch` code [14], implementing the coincidence lattice method [15, 16]. The lattice constants of CrSBr within the heterostructure are $a = 3.557$ Å and $b = 4.742$ Å, in close agreement with experimental values [17, 18], while the MoSe$_2$ layer is kept unstrained with a lattice constant of 3.288 Å [19]. Therefore, the individual monolayers are barely strained in our heterostructure and we should be able to reliably extract band offsets as well as proximity exchange effets. In order to simulate quasi-2D systems, we add a vacuum of about 18 Å to avoid interactions between periodic images in our slab geometry. The resulting heterostructure is shown in Fig. S8.

Computational Details

The electronic structure calculations and structural relaxations of the MoSe$_2$/CrSBr heterostructure is performed by DFT [20] with `Quantum ESPRESSO` [21]. Self-consistent calculations are carried out with a k-point sampling of $12 \times 18 \times 1$. We perform open shell

calculations that provide the spin-polarized ground state of the CrSBr monolayer. A Hubbard parameter of $U = 2.0$ eV is used for Cr d-orbitals. We use an energy cutoff for charge density of 560 Ry and the kinetic energy cutoff for wavefunctions is 70 Ry for the (scalar) relativistic pseudopotentials with the projector augmented wave method [22] with the Perdew-Burke-Ernzerhof exchange correlation functional [23]. For the relaxation of the heterostructures, we add DFT-D2 vdW corrections [24–26] and use quasi-Newton algorithm based on trust radius procedure. To get proper interlayer distances and to capture possible moiré reconstructions, we allow all atoms to move freely within the heterostructure geometry during relaxation. Relaxation is performed until every component of each force is reduced below 2×10^{-4} [Ry/a_0], where a_0 is the Bohr radius.

Results

Without Spin-Orbit Coupling, Collinear Case

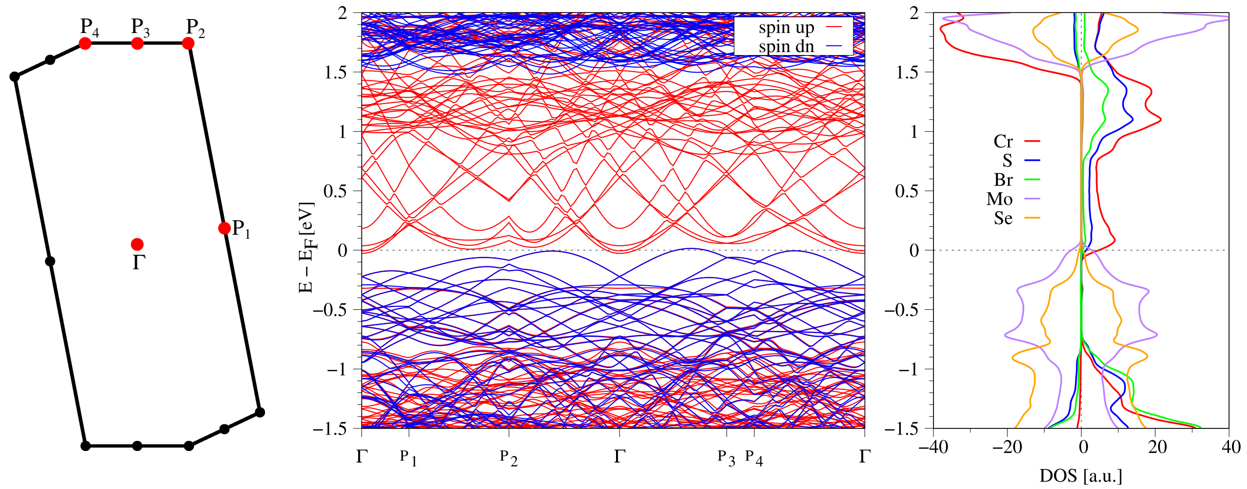

FIG. S9. — **Band Structure, Billouin Zone and density of states of the $MoSe_2$/CrSBr heterostructure.** Left: The Brillouin zone of the heterostructure, where we indicate high-symmetry points, along which the bands are calculated. Middle: DFT-calculated band structure of the $MoSe_2$/CrSBr heterostructure. Red (blue) lines correspond to spin up (down). Right: The corresponding spin and atom resolved density of states. Positive (negative) DOS is for spin up (down).

First, we discuss the first-principles results, where spin-orbit coupling is excluded from

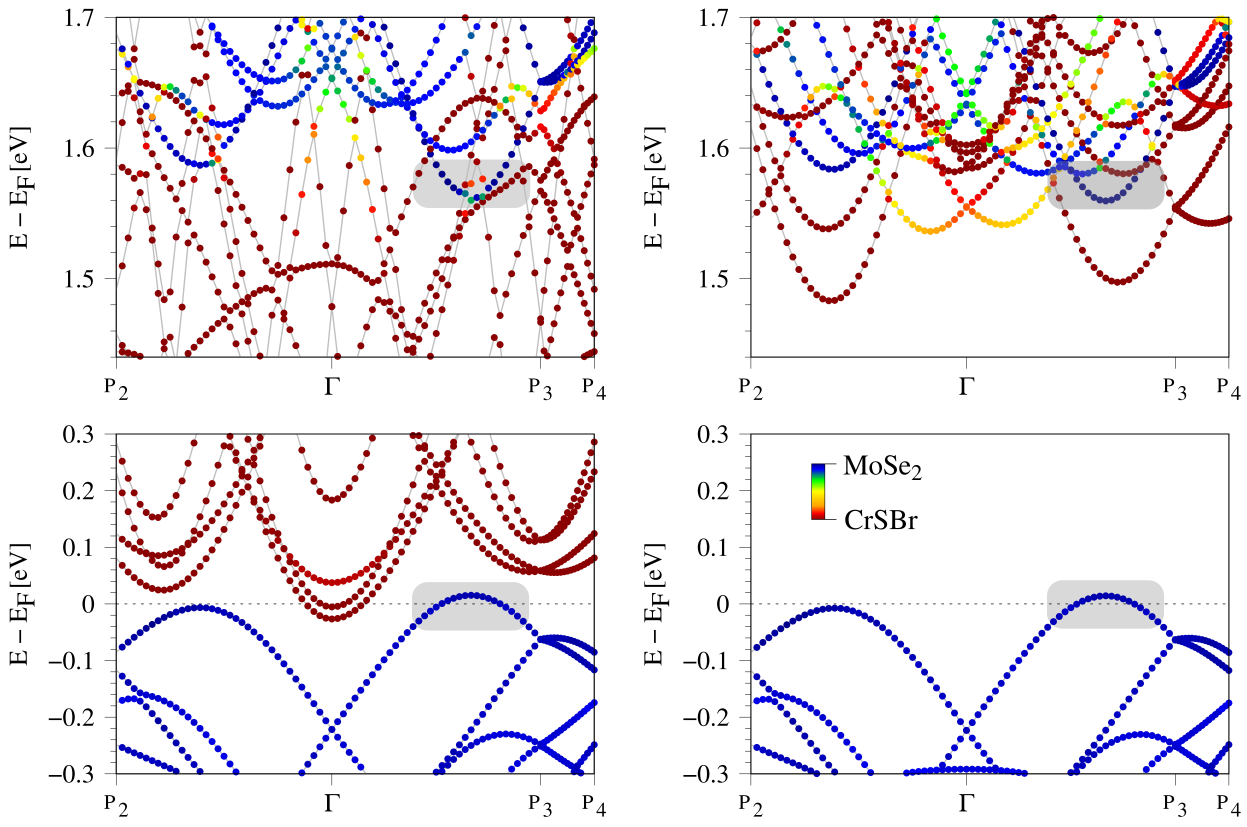

FIG. S10. — **Spin-resolved projected band structure in the vicinity of the $MoSe_2$ band edges.** Left/Right correspond to spin up/down channels. The color code represents the projection on the different monolayers. The $MoSe_2$ band edges are highlighted with grey boxes.

the calculations and the magnetization direction is fixed to be collinear with the z-axis (transverse to the interface). In Fig. S9 we show the calculated band structure and density of states of the $MoSe_2$/CrSBr heterostructure. We find that the heterostructure forms a type III band alignment, as the $MoSe_2$ valence band edge is about 15 meV above the Fermi level and the CrSBr conduction band edge is about 27 meV below the Fermi level.

We can estimate the hole density in the $MoSe_2$, by assuming parabolic bands for the $MoSe_2$ valence band edge, employing an effective mass of about 0.6m_e [27], and using the density of states in two dimensions for spin-degenerate bands $m^*/(\pi\hbar^2)$. The resulting hole density is $n = 3.76 \times 10^{12}$ cm^{-2}. However, one has to take into account the valley-degree of freedom, such that the hole density needs to be multiplied by a factor of 2, as we have this number of valleys in the Brillouin zone.

Detail views on the dispersion are shown in Fig. S10. The band edges of the $MoSe_2$

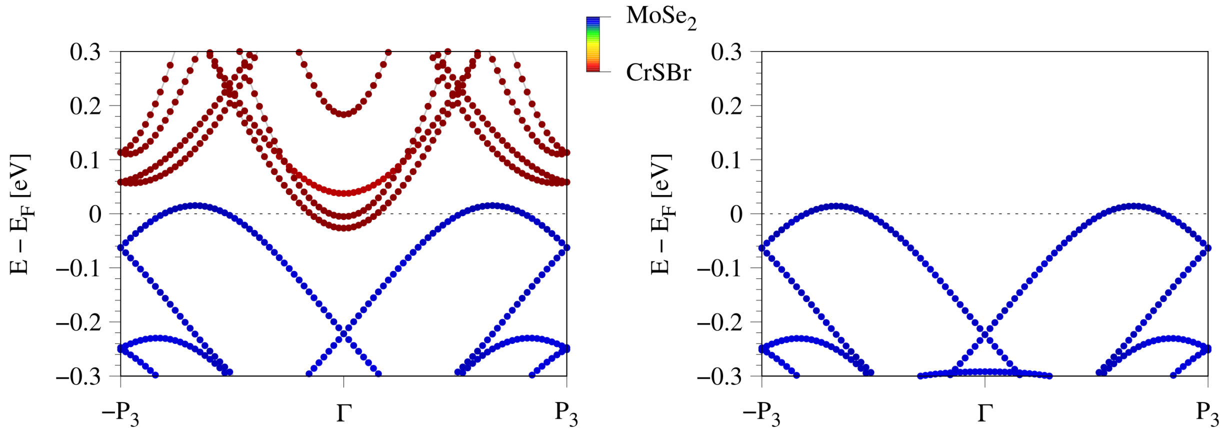

FIG. S11. — **Spin-resolved projected band structure near the Fermi level.** Left/Right correspond to spin up/down channels. The color code represents the projection on the different monolayers. The Brillouin Zone path covers the relevant $MoSe_2$ valleys K and K' (in our case folded towards P_3 and -P_3). The valleys are energetically and spin degenerate.

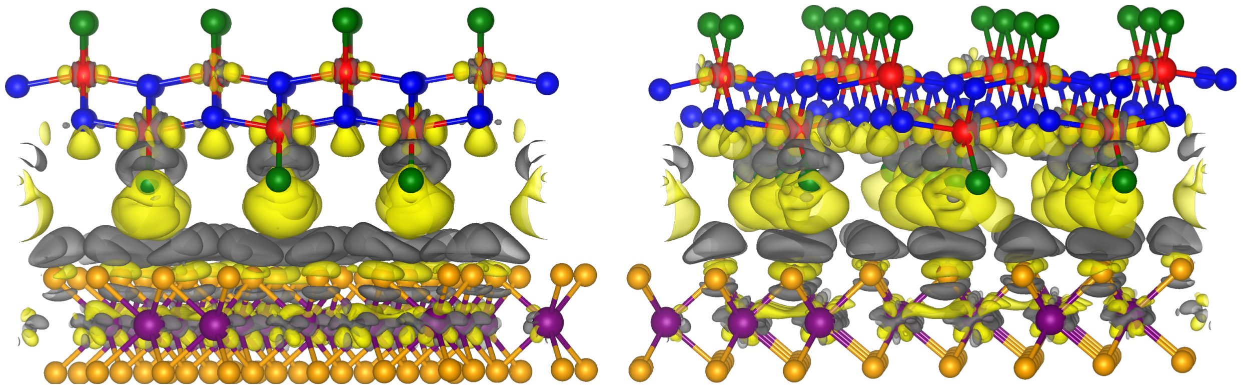

FIG. S12. — **DFT-calculated charge transfer.** The charge transfer is calculated by subtracting the monolayer charge densities from the heterostructure one. The colors yellow and gray correspond to gain and loss of charge. The isovalue is set to 1×10^{-4} e/Å^3.

are nicely preserved within the heterostructure. However, the conduction bands of $MoSe_2$ strongly hybridize with the substrate states, opening an efficient charge transfer channel when electron-hole pairs are optically excited. Additionally, the $MoSe_2$ valence band edge, which is folded at the Γ-P_3 line (highlighted in Fig. S10), is split by about 0.95 meV (the spin down band is at lower energies) due to proximity-induced exchange coupling. More precisely, the spin up (spin down) band edge of the $MoSe_2$ is about 15.2 (14.2) meV above the Fermi level, translating to a spin polarization of the hole density of about 6.5%. Looking

at both $MoSe_2$ valleys K and K', we find them to be energetically and spin degenerate, see Fig. S11, because of the absence of spin-orbit interaction.

In order to visualize the charge transfer, we have calculated the total charge densities of the heterostructure and the isolated monolayers. By subtracting the monolayer charge densities from the heterostructure one, the charge transfer (gain or loss) can be visualized. The result is shown in Fig. S12. We find that the overall charge transfer is strongly localized around the interface, since the isosurfaces are mainly localized around interfacial Br and Se atoms.

With Spin-Orbit Coupling, Noncollinear Case

To get a more realistic description of the heterostructure dispersion, we consider noncollinear magnetism and spin-orbit coupling in the calculations. The reason is that spin-orbit coupling is utterly important for the description of $MoSe_2$. In addition, CrSBr shows in-plane magnetism with the easy axis along the b lattice vector [28]. In our case, see Fig. S8, the b lattice vector of CrSBr, within the heterostructure, is tilted by about 46° with respect to the x-direction (the a heterostructure lattice vector). Due to the type-III band alignment of the heterostructure, see Fig. S9, we expect charge transfer between in-plane spin-polarized carriers of the CrSBr conduction band edge and out-of-plane spin-polarized carriers of the $MoSe_2$ valence band edge.

The full band structure, showing spin and layer projections is shown in Fig. S13. Similar to above, the heterostructure remains in the type III band alignment. However, due to the spin-orbit coupling, the $MoSe_2$ valence band edges strongly split. In addition, the $MoSe_2$ band edges and their s_z spin character is nicely preserved. Zooming in on the valence band edges of $MoSe_2$ for the two valleys K and K' (in our case folded towards P_3 and -P_3), see Fig. S14, we find them about 37.4 meV and 43.6 meV above the Fermi level, resulting in a splitting of about 6 meV. The CSB conduction band edge is about 33.6 meV below the Fermi level. The hole densities from K and K' valleys of $MoSe_2$, are $n = 4.69 \times 10^{12}$ cm^{-2} and $n = 5.46 \times 10^{12}$ cm^{-2} correspondingly, resulting in a total density of $n = 10.15 \times 10^{12}$ cm^{-2} Due to the spin-valley locking, the hole density is spin polarized with a polarization of about 7.6%. Comparing to the case of z-collinear magnetism without spin-orbit coupling (see above), we find similar values for the polarization and the hole density. The conduction

band edges of $MoSe_2$ are buried within the CSB conduction band manifold. In fact, there is significant hybridization present between the layers, as we find from the projected band structure.

Analyzing the layer resolved averaged magnetic moments, as summarized in Table I, we find the CSB magnetism to be in-plane and directed parallel to the b-axis as expected. The $MoSe_2$ magnetic moments result from the proximity-induced exchange coupling. The induced moments are generally small, and are almost already absent in the topmost Se-layer, due to the short-rangeness of proximity coupling. Additionally, the induced magnetism in $MoSe_2$ also shows a preferential in-plane component, directed antiparallel to the Cr magnetic moments of CSB, but parallel to the interfacial Br magnetic moments. This indicates that the magnetism is transferred through hybridizing wavefunctions at the interface across the van der Waals gap.

In Fig. S15 we show the calculated orthogonal spin textures of the $MoSe_2$ K/K'-valley and CSB Γ-valley band edges near the Fermi level, in order to find out about the spin-texture of the transferred charge carriers. The K/K' valleys show a dominant out-of-plane s_z character, with small in-plane $s_{||} = \sqrt{s_x^2 + s_y^2}$ contributions. For the K (K') valley, the ratio $s_{||}/s_z$ is in the range of 2.5–4.0$\times10^{-3}$ (2.2–5.3$\times10^{-3}$). The in-plane spin components, at the $MoSe_2$ K (K') valley edge, have an angle of around 42° (35°) with respect to the x-axis, almost collinear to the magnetic easy-axis of CrSBr. For the CrSBr Γ-Valley, the in-plane spin character dominates. The in-plane spins are collinear with the CrSBr magnetization, having an angle of 46° with respect to the x-axis. The ratio $s_z/s_{||}$ near the valley edge is about 2.4$\times10^{-4}$. This indicates, that the $MoSe_2$- and CrSBr-like bands retain their spin character in the heterobilayer to a large extent.

Concluding discussion about the proximity-induced exchange field

In fact, most studies have considered either an out-of-plane (perpendicular to the TMDC) magnetic field, or a magnetic substrate with out-of-plane magnetism. In the case of an external magnetic field, the Zeeman energy leads to the shift of the band edges, opposite in opposite valleys due to spin-valley locking in the TMDC. A simple picture, for the proximity effect in our $MoSe_2$/CrSBr bilayer, could be to view the magnetic substrate as a source of an exchange field that acts in a similar way as an external magnetic field. However, the

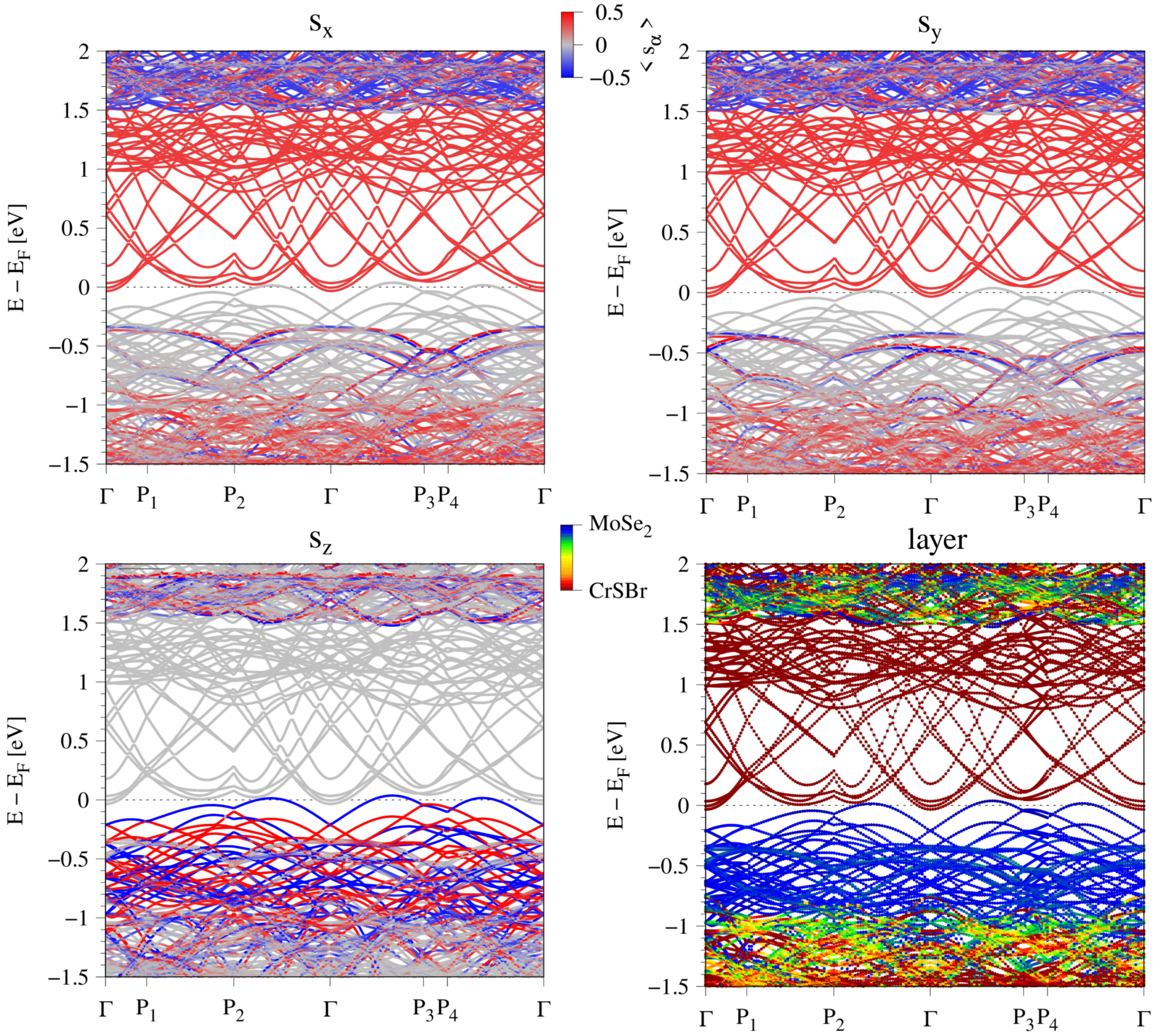

FIG. S13. DFT-calculated band structure of the $MoSe_2$/CrSBr heterostructure, with spin-orbit coupling and including the noncollinear magnetism. We show the bands with spin (s_α) and layer projections.

mechanism behind is the hybridization of the monolayer wavefunctions across the van der Waals gap. That means, analyzing the wavefunctions of the TMDC band edges at K/K', we find a small amount of CrSBr states there (approx. 1-2%). Consequently, a small amount of exchange coupling is effectively transferred to the $MoSe_2$ layer, which leads to the valley splitting. This is called the bare proximity effect. Additionally, in the heterostructure dispersion, bands of the individual monolayers that show significant coupling, can strongly anticross, further renormalizing the valley splitting. This is what we call a hybridization

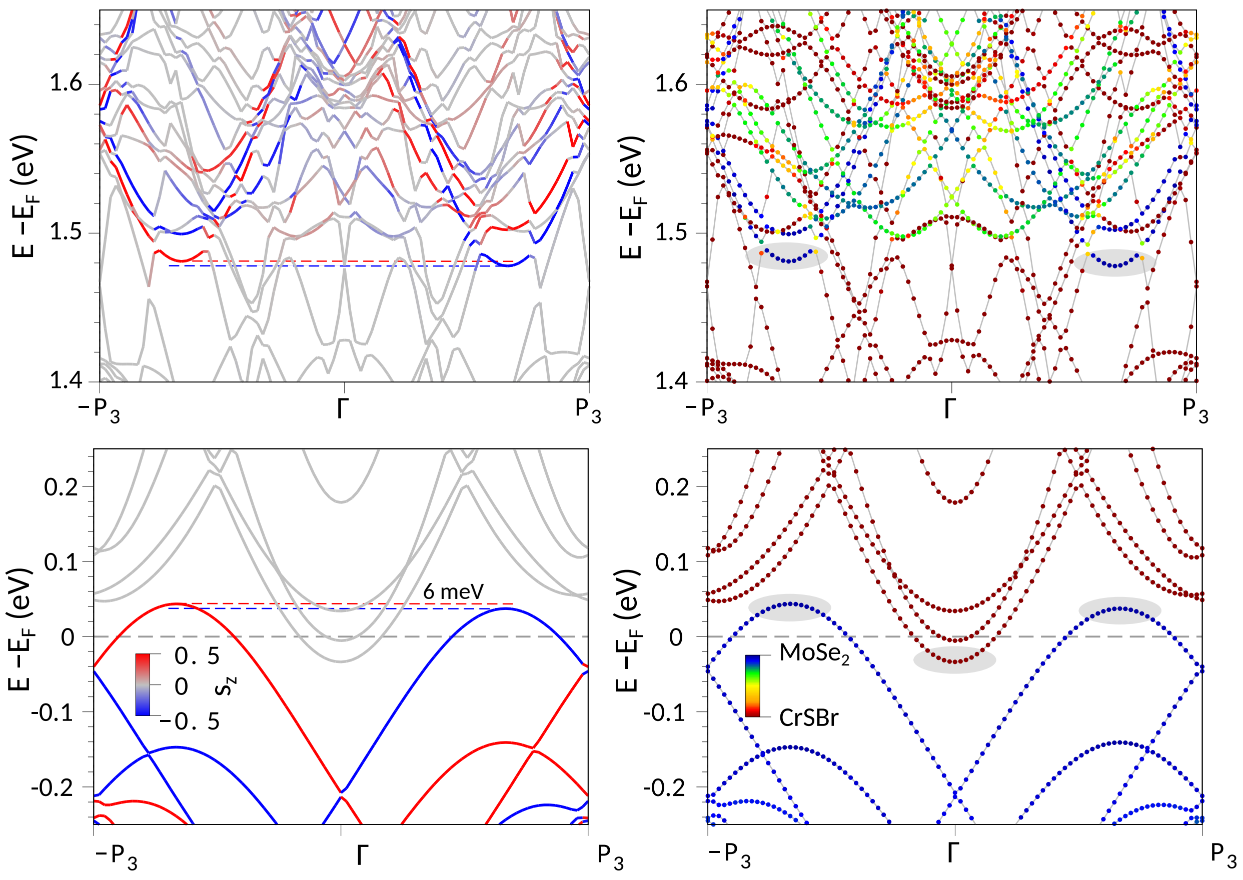

FIG. S14. DFT-calculated band structure of the $MoSe_2$/CrSBr heterostructure near the Fermi level, including spin-orbit coupling and the noncollinear magnetism. We show the bands with s_z and layer projections. The Brillouin Zone path covers the relevant $MoSe_2$ valleys K and K' (in our case folded towards P_3 and -P_3). The relevant band edges are highlighted with grey shaded areas. The corresponding spin-orbit fields are shown in Fig. S15.

(anticrossing) dependent renormalization of the proximity effect, which has been observed in $MoSe_2$/$CrBr_3$ [29] or graphene/hBN/Co [30]. In particular in the $MoSe_2$/$CrBr_3$ case, the energetic alignment of the monolayer bands (and also their spin polarization) significantly alters the valley splitting.

This picture matches very well with our DFT calculation results. In the case without SOC, the band hybridization (anticrossing) is suppressed, as opposite spins cannot anticross, leading to 1 meV valley splitting. Once SOC is taken into account, bands of different spins are also allowed to anticross, leading to a renormalization of the valley splitting, up to 6

TABLE I. Layer resolved averaged magnetic moments (MM) in units μ_B and magnetization angles ϑ (z-axis) and φ (x-axis). In the case of collinear magnetism, the magnetic moments are parallel (+) or antiparallel (-) to the z-axis.

	no SOC	SOC		
	z-collinear	noncollinear		
	MM	MM	ϑ	φ
Se	0.00000	0.00021	61.3884	48.3301
Mo	-0.00181	0.00235	89.1369	-88.5649
Se	-0.00138	0.00156	79.5134	-132.2449
Br	-0.03651	0.03643	89.7367	-134.3114
Cr	2.86493	2.86789	89.9998	45.9999
S	-0.16783	0.16844	89.9991	-133.9992
S	-0.16924	0.17029	89.9972	-134.0026
Cr	2.85314	2.85570	90.0000	45.9988
Br	-0.03625	0.03651	90.0358	-134.2959

meV in our case. Note that the band hybridization can occur several 100 meV away from the relevant TMDC band edges and still lead to significant splitting of the TMDC band edges, as demonstrated for Dirac states in graphene [31].

Certainly, the in-plane magnetism of CrSBr is not predetermined to induce such a large valley splitting. However, from our band-structure calculations we find the above picture to be conclusive, as significant hybridization is shown in Figs. S13 and S14, where $MoSe_2$ bands pick up also an in-plane spin character. This is also consistent with the induced magnetic moments in $MoSe_2$, following the in-plane magnetism of CrSBr, and also with the spin-orbit fields of the TMDC band edges showing in-plane spin components. Also, keep in mind that the proximity effect is mutual, i.e., CrSBr is influenced by $MoSe_2$, providing strong Ising SOC. In conclusion, to grasp the full details of proximity effects is not a straightforward task.

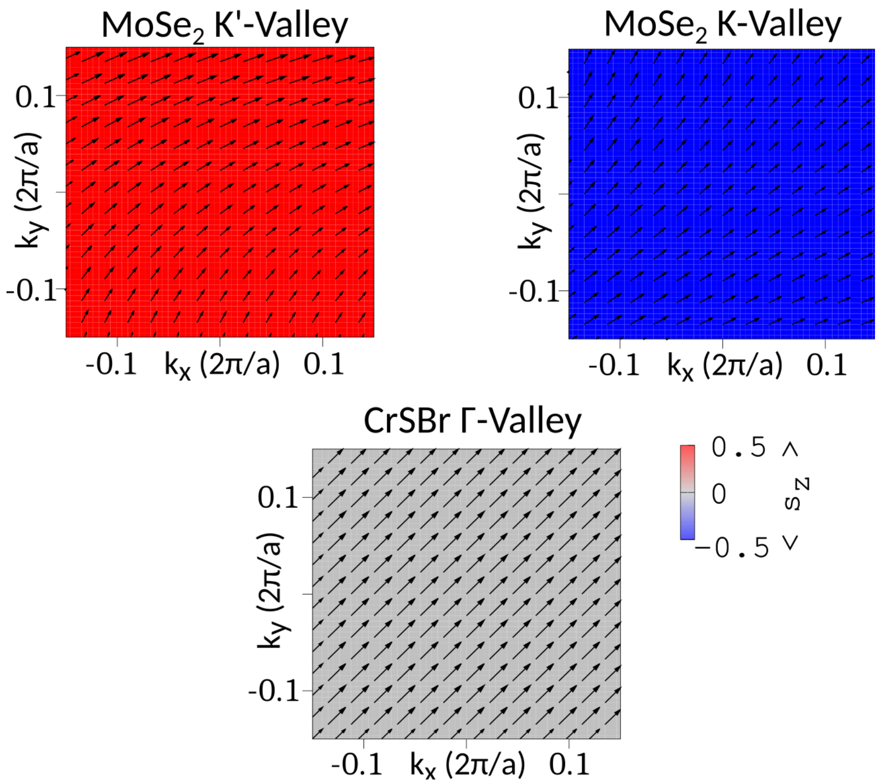

FIG. S15. Calculated spin textures in the vicinity of relevant band edges near the Fermi level. The arrows correspond to in-plane spins, while the color corresponds to the out-of-plane spin.

[1] J. J. Baumberg, D. D. Awschalom, N. Samarth, H. Luo, and J. K. Furdyna, Spin beats and dynamical magnetization in quantum structures, Phys. Rev. Lett. **72**, 717 (1994). https://doi.org/10.1103/PhysRevLett.72.717

[2] S. Raiber, P. E. Faria Junior, D. Falter, S. Feldl, P. Marzena, K. Watanabe, T. Taniguchi, J. Fabian, and C. Schüller, Ultrafast pseudospin quantum beats in multilayer WSe_2 and $MoSe_2$, Nat. Commun. **13**, 4997 (2022). doi.org/10.1038/s41467-02232534-3

[3] C. Robert, D. Lagarde, F. Cadiz, G. Wang, B. Lassagne, T. Amand, A. Balocchi, P. Renucci, S. Tongay, B. Urbaszek, and X. Marie, Exciton radiative lifetime in transition metal dichalcogenide monolayers, Phys. Rev. B **93**, 205423 (2016).

[4] H. Tornatzky, A.-M. Kaulitz, and J. Maultzsch, Resonance Profiles of Valley Polarization in Single-Layer MoS_2 and $MoSe_2$, Phys. Rev. Lett. **121**, 167401 (2018). doi.org/10.1103/PhysRevLett.121.167401

[5] M. Z. Maialle, E. A. de Andrada E Silva, and L. J. Sham, Exciton spin dynamics in quantum wells, Phys. Rev. B **47**, 15776 (1993).

[6] M. M. Glazov, T. Amand, X. Marie, D. Lagarde, L. Bouet, and B. Urbaszek, Exciton fine structure and spin decoherence in monolayers of transition metal dichalcogenides, Phys. Rev. B **89**, 201302(R) (2014).

[7] T. Yu and M. W. Wu, Valley depolarization due to intervalley and intravalley electron-hole exchange interactions in monolayer MoS_2, Phys. Rev. B **89**, 205303 (2014).

[8] C. R. Zhu, K. Zhang, M. Glazov, B. Urbaszek, T. Amand, Z. W. Ji, B. L. Liu, and X. Marie, Exciton valley dynamics probed by Kerr rotation in WSe_2 monolayers, Phys. Rev. B **90**, 161302(R) (2014).

[9] K. Hao, L. Xu, P. Nagler, A. Singh, K. Tran, C. K. Dass, C. Schüller, T. Korn, X. Li, and G. Moody, Coherent and Incoherent Coupling Dynamics between Neutral and Charged Excitons in Monolayer $MoSe_2$, Nano Lett. **16**, 5109 (2016).

[10] Q. Zhang, H. Sun, J. Tang, X. Dai, Z. Wang, and C.-Z. Ning, Prolonging valley polarization lifetime through gate-controlled exciton-to-trion conversion in monolayer molybdenum ditelluride, Nat. Commun. **13**, 4101 (2022). doi.org/10.1038/s41467-022-31672-y

[11] Y. J. Bae, J. Wang, A. Scheie, J. Xu, D. G. Chica, G. Diederich, J. Cenker, M. Ziebel, Y.

Bai, H. Ren, C. Dean, M. Delor, X. Xu, X. Roy, A. D. Kent, and X. Zhu, Exciton-coupled coherent magnons in a 2D semiconductor, Nature **609**, 282, (2022).

[12] G. M. Diederich, J. Cenker, Y. Ren, J. Fonseca, D. G. Chica, Y. J. Bae, X. Zhu, X. Roy, T. Cao, D. Xiao, and X. Xu, Tunable interaction between excitons and hybridized magnons in a layered semiconductor, Nat Nanotechnol. **18**, 23, (2021).

[13] S. R. Bahn and K. W. Jacobsen, An object-oriented scripting interface to a legacy electronic structure code, Comput. Sci. Eng. **4**, 56 (2002).

[14] Predrag Lazic, Cellmatch: Combining two unit cells into a common supercell with minimal strain, Computer Physics Communications **197**, 324 (2015).

[15] D. S. Koda, F. Bechstedt, M. Marques, and L. K. Teles, Coincidence lattices of 2d crystals: heterostructure predictions and applications, The Journal of Physical Chemistry C **120**, 10895 (2016).

[16] S. Carr, S. Fang, and E. Kaxiras, Electronic-structure methods for twisted moire layers, Nature Reviews Materials **5**, 748 (2020).

[17] The Materials Project, Materials data on CrSBr by materials project, (2020), DOI: https://doi.org/10.17188/1199151 (date of access 10/11/2024).

[18] Johannes Beck, Über Chalkogenidhalogenide des Chroms Synthese, Kristallstruktur und Magnetismus von Chromsulfidbromid, CrSBr," Zeitschrift für anorganische und allgemeine Chemie **585**, 157 (1990).

[19] W. J. Schutte, J. L. De Boer, and F. Jellinek, Crystal structures of tungsten disulfide and diselenide, Journal of Solid State Chemistry **70**, 207 (1987).

[20] P. Hohenberg and W. Kohn, Inhomogeneous electron gas, Phys. Rev. **136**, B864 (1964).

[21] P. Giannozzi and et al., Quantum espresso: a modular and open-source software project for quantum simulations of materials, J. Phys.: Cond. Mat. **21**, 395502 (2009).

[22] G. Kresse and D. Joubert, From ultrasoft pseudopotentials to the projector augmented-wave method, Phys. Rev. B **59**, 1758 (1999).

[23] J. P. Perdew, K.n Burke, and M. Ernzerhof, Generalized gradient approximation made simple, Phys. Rev. Lett. **77**, 3865 (1996).

[24] S. Grimme, Semiempirical GGA-type density functional constructed with a long-range dispersion correction, J. Comput. Chem. **27**, 1787 (2006).

[25] S. Grimme, J. Antony, S. Ehrlich, and H. Krieg, A consistent and accurate ab initio

parametrization of density functional dispersion correction (DFT-D) for the 94 elements H-Pu, J. Chem. Phys. **132**, 154104 (2010).

[26] V. Barone, M. Casarin, D. Forrer, M. Pavone, M. Sambi, and A. Vittadini, Role and effective treatment of dispersive forces in materials: Polyethylene and graphite crystals as test cases, J. Comput. Chem. **30**, 934 (2009).

[27] A. Kormányos, G. Burkard, M. Gmitra, J. Fabian, V. Zólyomi, N. D. Drummond, V. Fal'ko, k·p theory for two-dimensional transition metal dichalcogenide semiconductors, 2D Materials **2**, 022001 (2015).

[28] J. Klein, B. Pingault, M. Florian, M.-C. Heißenbüttel, A. Steinhoff, Z. Song, K. Torres, F. Dirnberger, J. B. Curtis, M. Weile, A. Penn, T. Deilmann, R. Dana, R. Bushati, J. Quan, J. Luxa, Z. Sofer, A. Alù, V. M. Menon, U. Wurstbauer, M. Rohlfing, P. Narang, M. Lončar, F. M. Ross, The Bulk van der Waals Layered Magnet CrSBr is a Quasi-1D Material, ACS Nano **17**, 5316 (2023).

[29] J. Choi, C. Lane, J.-X. Zhu, and S. A. Crooker, Asymmetric magnetic proximity interactions in $MoSe_2/CrBr_3$ van der Waals heterostructures, Nat. Materials **22**, 305 (2023).

[30] K. Zollner, M. Gmitra, T. Frank, and J. Fabian, Theory of proximity-induced exchange coupling in graphene on hBN/(Co, Ni), Phys. Rev. B **94**, 155441 (2016).

[31] K. Zollner and J. Fabian, Engineering Proximity Exchange by Twisting: Reversal of Ferromagnetic and Emergence of Antiferromagnetic Dirac Bands in Graphene/$Cr_2Ge_2Te_6$, Phys. Rev. Lett. **128**, 106401 (2022).

[32] C. Serati de Brito, P. E. Faria Junior, T. S. Ghiasi, J. Ingla-Aynés, C. R. Rabahi, C. Cavalini, F. Dirnberger, S. Mañas-Valero, K. Watanabe, T. Taniguchi, K. Zollner, J. Fabian, C. Schüller, H. S. J. van der Zant, and Y. Galvão Gobato, Charge Transfer and Asymmetric Coupling of $MoSe_2$ Valleys to the Magnetic Order of CrSBr, Nano Lett. **23**, 11073 (2023).
